# Endothelial monocarboxylate transporter 1 drives atherosclerosis via a lactate/NADH/CtBP‐mediated transrepression pathway

**DOI:** 10.1002/mco2.70089

**Published:** 2025-02-13

**Authors:** Zou Li, Shuai Guo, Kaixiang Cao, Yuxi Duan, Yuan Zhao, Yuting Zhang, Shihui Yu, Zaixia Bai, Runfa Yu, Yixin Chen, Ziling Li, Shuqi Huang, Mingchuan Song, Cailing Wang, Wenzhong Hou, Jun He, Bin Yang, Yiming Xu

**Affiliations:** ^1^ Department of Emergency The Second Affiliated Hospital, Guangzhou Medical University Guangzhou China; ^2^ Department of Physiology, School of Basic Medical Sciences Guangzhou Medical University Guangzhou China; ^3^ Department of Nephrology, the Fourth Affiliated Hospital Guangzhou Medical University Guangzhou China; ^4^ Department of Cerebrovascular Disease The Affiliated Qingyuan Hospital (Qingyuan People's Hospital) Guangzhou Medical University Qingyuan China; ^5^ Department of Rehabilitation Center The First Affiliated Hospital of Guangzhou University of Chinese Medicine Guangzhou China

**Keywords:** atherosclerosis, endothelial cell, FOXP1, lactate, MCT1

## Abstract

The accumulation of lactate in tissue microenvironments is associated with atherosclerosis, but its precise role in atherogenesis remains largely unknown. This study demonstrated that lactate accumulation in aortic tissues and blood is correlated with increased monocarboxylate transporter 1 (Mct1) expression in endothelial cells (ECs) within atherosclerotic plaques. Lactate uptake via Mct1 triggers an inflammatory response in ECs. The administration of endothelial‐targeting nanoparticles containing siRNA against *Mct1* reduces endothelial inflammation and atherogenesis in *Apoe^−/−^* mice. Mechanistic studies revealed that the conversion of lactate to pyruvate, along with NADH production and oligomerization of the NADH‐sensitive transcriptional corepressor C‐terminal binding protein 1 (CtBP1), is necessary for the proinflammatory effects of lactate. Monomeric CtBP1 interacts with the transcriptional repressor forkhead box P1 (FOXP1) to suppress endothelial adhesion molecule expression. However, NADH‐induced oligomerization of CtBP1 prevents its binding to FOXP1, significantly reducing FOXP1‐mediated transrepression of endothelial adhesion molecules. Moreover, silencing *Foxp1* in ECs negates the atheroprotective effect of endothelial *Mct1* knockdown in *Apoe^−/−^* mice. These findings suggest that lactate/MCT1‐induced epigenetic reprogramming represents a potential therapeutic target in atherosclerosis.

## Graphical abstract

In this study, we identified the buildup of lactate within the tissue microenvironment in the context of atherosclerosis. Our findings indicate that MCT1‐mediated lactate uptake enhances endothelial activation by modulating FOXP1 transrepressive activity through a CtBP1 oligomerization‐dependent mechanism, thereby inducing atherosclerotic lesion formation. Furthermore, the use of endothelial‐targeting nanoparticles loaded with siRNA against *Mct1* was shown to mitigate endothelial inflammation and atherogenesis in *Apoe* ^−/−^ mice.

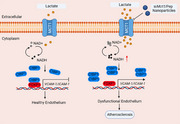

## 1 INTRODUCTION

Atherosclerosis, a chronic inflammatory condition leading to heart and brain ischemia, is the primary global cause of mortality and morbidity.^1^ Endothelial dysfunction is the initiating event in the development of atherosclerosis.^2^ In the context of atherosclerosis, vascular endothelial cells (ECs) undergo excessive activation due to factors such as irregular blood flow, lipid accumulation in the vessel wall, and exposure to inflammatory agents.^3^, ^4^, ^5^ These dysfunctional ECs exhibit high levels of adhesion molecules, such as vascular cell adhesion molecule‐1 (VCAM‐1) and intracellular adhesion molecule‐1 (ICAM‐1), which orchestrate the infiltration and retention of inflammatory cells within evolving atherosclerotic plaques. Therefore, understanding the EC response to the inflammatory environment driving plaque development is crucial for effective atherosclerosis management.

Lactate, once considered merely a byproduct of glycolysis, has been redefined by recent studies as a molecule with broad physiological and pathological implications. It is currently recognized that lactate serves as a primary carbon source for the tricarboxylic acid cycle in all tissues, except the brain, and even surpasses glucose.^6^ Under normal conditions, lactate concentrations in blood and healthy tissues range from approximately 1.5 to 3 mM. However, in inflamed tissues such as tumor microenvironments, arthritic joints, and atherosclerotic plaques, these concentrations can increase to between 10 and 40 mM. Clinical studies have shown an increase in blood lactate levels in carotid atherosclerosis patients and established a strong correlation with plaque burden, independent of traditional cardiovascular risk factors.^7^, ^8^ Lactate crosses the cell membranes of various tissues using a facilitated monocarboxylate transport system, which includes proton–lactate symporters (such as MCT1‐4) and sodium‐dependent transporters (such as SLC5A8 and SLC5A12). Among these, MCT1 has been identified as the primary facilitator of lactate uptake in ECs within the context of various inflammatory diseases and tumors.^9^, ^10^

Recent research has shown that lactate significantly impacts vascular function by triggering the dedifferentiation of vascular smooth muscle cells (VSMCs).^11^, ^12^ In ECs within neoplastic tissues, lactate activates the NF‐κB signaling pathway, thereby regulating the inflammatory response.^9^ Elevated serum lactate levels under septic conditions has been shown to cause endothelial barrier dysfunction, increasing vascular permeability and worsening organ damage.^13^ Additionally, lactate stimulates endothelial‐to‐mesenchymal transition (EndoMT) following myocardial infarction.^10^ EndoMT promotes endothelial dysfunction and the onset of atherosclerosis,^14^ suggesting that lactate may play a role in promoting endothelial dysfunction during the development of atherosclerosis. However, the role of lactate in endothelial dysfunction in atherosclerosis remains unclear and requires further experimental validation.

In this study, we revealed an unrecognized role of lactate in driving endothelial inflammation during atherosclerosis. Our findings indicate that lactate uptake into ECs via MCT1 increases vascular inflammation by upregulating VCAM‐1 and ICAM‐1, thereby contributing to atherosclerosis. This involves NADH generation through lactate oxidation and the subsequent oligomerization of the NADH‐sensitive transcriptional corepressor CtBP1. Additionally, FOXP1 transcriptionally suppresses VCAM‐1 and ICAM‐1, with CtBP1 oligomerization influencing FOXP1 activity. Inhibiting MCT1 reduces lactate‐induced vascular inflammation and atherosclerosis in *Apoe^−/−^* mice, suggesting that the lactate‐dependent NADH/CtBP/FOXP1 pathway is a potential therapeutic target.

## 2 RESULTS

### 2.1 Lactate promotes vascular inflammation in atherosclerosis

**FIGURE 1:**
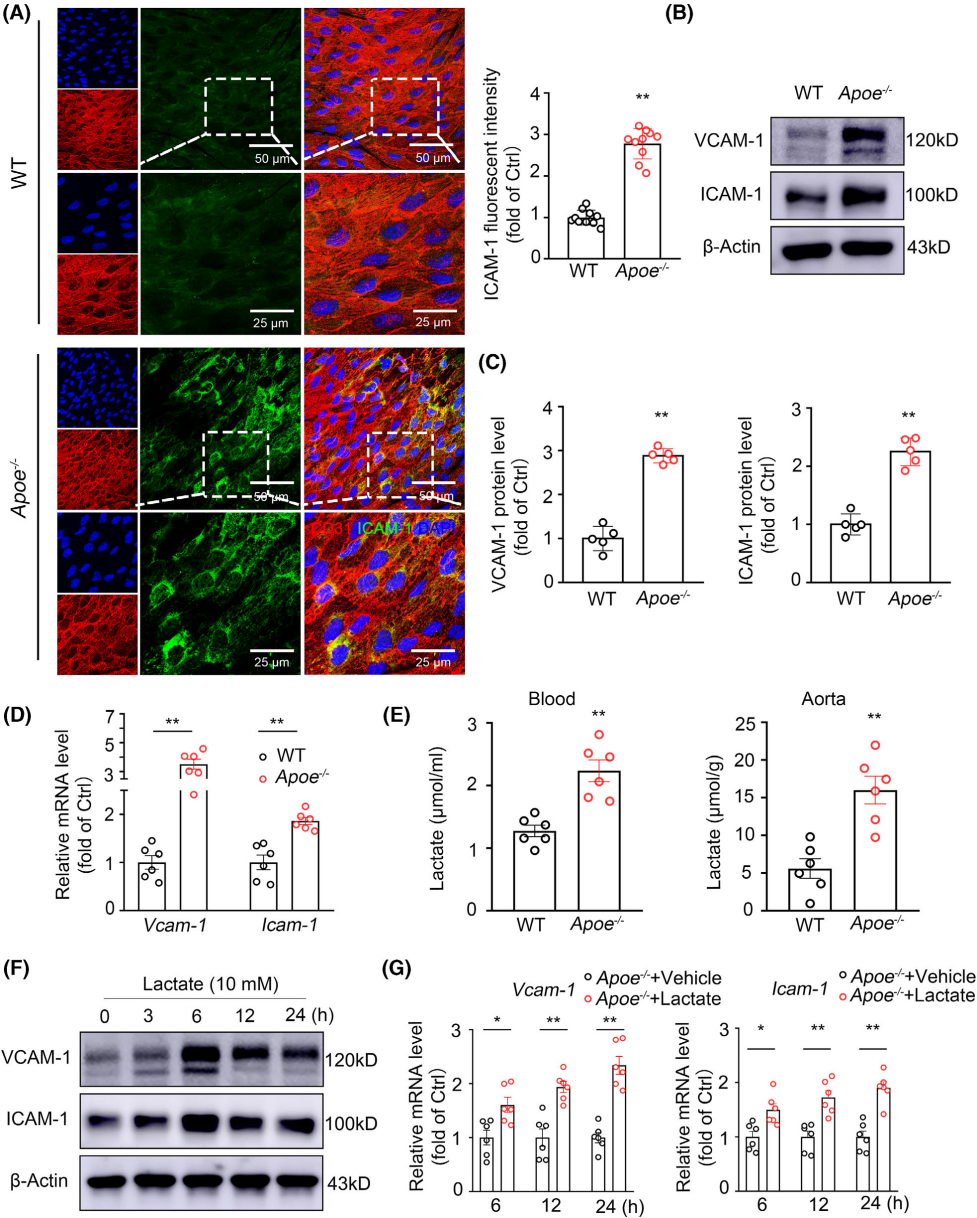
Lactate promotes vascular inflammation in atherosclerosis. (A) Representative images and quantification data of *en face* IF staining for Icam‐1 (green) in the aortic endothelium from wild‐type (WT) and *Apoe* ^−/−^ mice treated with WD for 1 month. The endothelium was visualized by CD31 staining (red). Nuclei were counterstained with DAPI (blue) (*n* = 10 mice per group). (B and C) Western blot analysis and quantification of protein levels of Vcam‐1 and Icam‐1 in MAECs isolated from WT and *Apoe* ^−/−^ mice (*n* = 5 mice per group). (D) Real‐time PCR analysis of the mRNA levels of *Vcam‐1* and *Icam‐1* genes in endothelial RNA obtained from aortas of 1‐month WD‐fed WT and *Apoe* ^−/−^ mice through flushing with TRIzol reagent (*n* = 6 mice per group). (E) The lactate levels in blood and aortas from 1‐month WD‐fed WT and *Apoe* ^−/−^ mice were measured by Lactate Assay Kit (*n* = 6 mice per group). (F) Western blot detection of VCAM‐1 and ICAM‐1 in HUVECs treated with lactate (10 mM) or vehicle for 0–24 h. (G) Real‐time PCR analysis of the mRNA levels of *Vcam‐1* and *Icam‐1* genes in endothelial RNA obtained from 1‐month WD‐fed *Apoe* ^−/−^ mice treated with lactate (0.5 g/kg body weight) or vehicle via i.p. injection for 6, 12, or 24 h. Endothelial RNA was extracted from mouse aortas through flushing with TRIzol reagent (*n* = 6 mice per group). Data were expressed as mean ± SEM. Statistical significance was assessed using an unpaired, two‐tailed Student's *t*‐test (A, C, D, E) or one‐way ANOVA with Tukey's post hoc test (G). Compared with the WT group, **p < 0.01 (A, C, D, E). Compared with the *Apoe* ^−/−^+Vehicle group, *p < 0.05 and **p < 0.01 (G).

To explore the connection between lactate and endothelial inflammation in atherosclerosis, we assessed Vcam‐1 and Icam‐1 expression in the aortic endothelium and measured lactate levels in the aortic tissues and blood of *Apoe* ^−/−^ mice and wild‐type (WT) mice that were fed a Western diet (WD) for 1 month. *En face* immunofluorescence staining of the thoracic aorta endothelium revealed significantly greater Icam‐1 expression in *Apoe^−/−^* mice than in WT mice (Figure 1A). Western blot analysis confirmed increased Icam‐1 and Vcam‐1 protein levels in the aortas of the *Apoe* ^−/−^ mice compared with those in the WT mice (Figure 1B,C), and the aortic ECs from the *Apoe* ^−/−^ mice had higher *Icam‐1* and *Vcam‐1* mRNA levels compared with those from the WT mice (Figure 1D). Lactate levels in aortic tissues and blood were also markedly greater in *Apoe* ^−/−^ mice than in WT mice (Figure 1E). To further explore the functional role of lactate in endothelial inflammation, human umbilical vein ECs (HUVECs) were treated with 10 mM lactate (adjusted to pH 6.8) for 0–24 h. Lactate treatment significantly increased ICAM‐1 and VCAM‐1 expression, which peaked at 6 h (Figure 1F). The upregulation of *VCAM‐1* and *ICAM‐1* expression by lactate treatment was further confirmed at the mRNA level (Figure S1A). To examine whether lactate promotes endothelial inflammation in vivo, we administered lactate (adjusted to pH 6.8, 0.5 g/kg body weight) to *Apoe* ^−/−^ mice via intraperitoneal injection and examined Vcam‐1 and Icam‐1 levels in the aortic endothelium. As depicted in Figure S1B, the serum lactate levels peaked approximately 6 h after injection and returned to baseline levels at approximately 6 days. In addition, the administration of lactate increased Vcam‐1 and Icam‐1 levels in the aortic endothelium (Figure 1G). Collectively, these data demonstrate that increasing lactate levels promote endothelial inflammation in the context of atherosclerosis.

### 2.2 MCT1 facilitates lactate uptake into ECs for vascular inflammation

**FIGURE 2:**
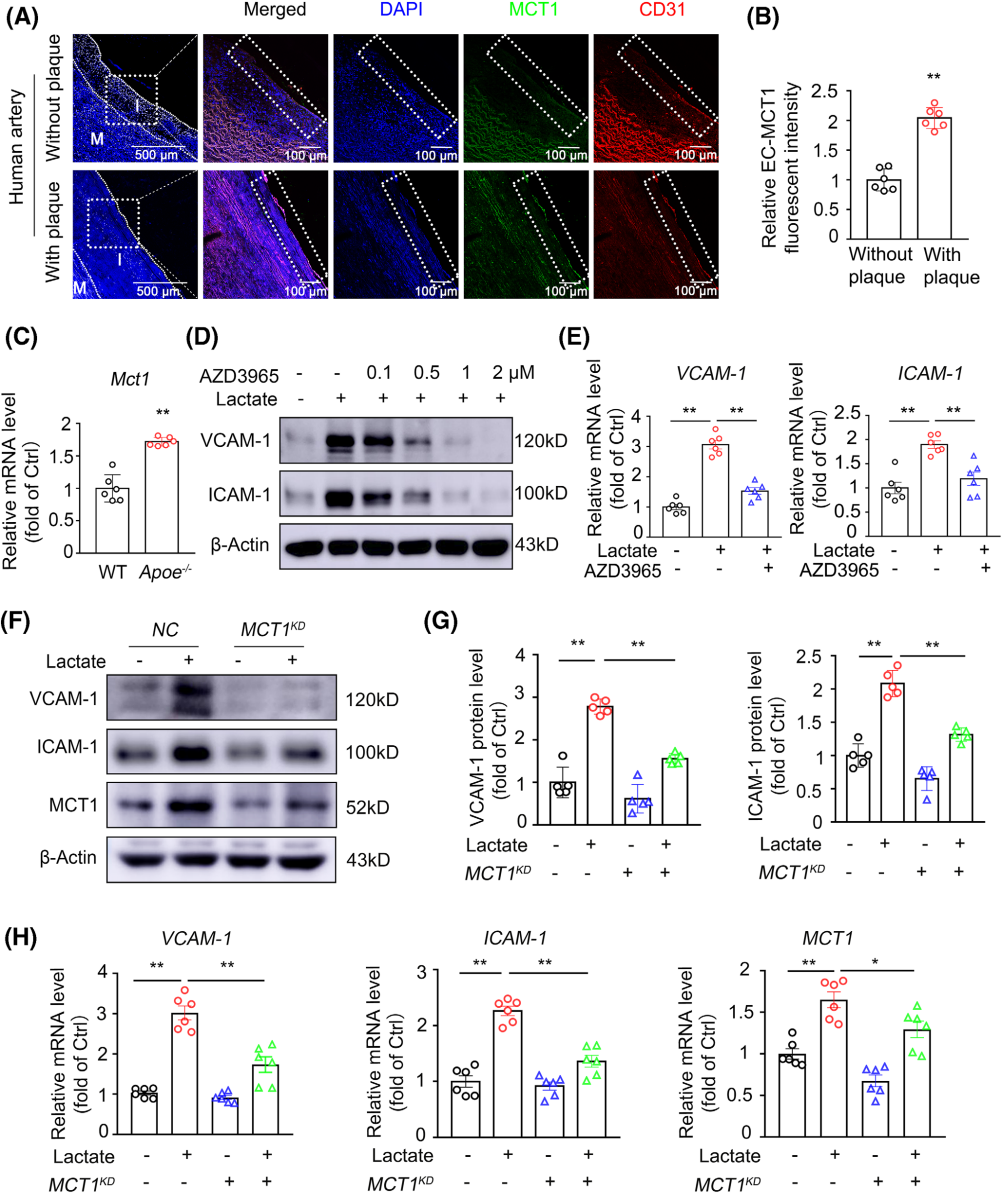
MCT1 facilitates lactate uptake into ECs for vascular inflammation. (A and B) Representative IF staining and quantification of MCT1 (green) in normal (upper panels) and stenotic (lower panels) human carotid artery specimens. The endothelium was visualized by CD31 staining (red). Nuclei were counterstained with DAPI (blue) (*n* = 6). (C) Real‐time PCR analysis of the mRNA levels of *Mct1* genes in endothelial RNA obtained from aortas of 1‐month WD‐fed WT and *Apoe* ^−/−^ mice through flushing with TRIzol reagent (*n* = 6 mice per group). (D) Western blot detection of VCAM‐1 and ICAM‐1 in HUVECs pretreated with the MCT1 inhibitor AZD3965 (500 nM) for 24 h and then stimulated with lactate (10 mM) or vehicle for 6 h (*n* = 3). (E) Real‐time PCR analysis of the mRNA levels of *VCAM‐1* and *ICAM‐1* genes in HUVECs pretreated with the MCT1 inhibitor AZD3965 (500 nM) for 24 h and then stimulated with lactate (10 mM) or vehicle for 3 h (*n* = 6). (F and G) Western blot detection and quantification of protein levels of VCAM‐1 and ICAM‐1 in *MCT1* KD or control HUVECs with lactate (10 mM) or vehicle stimulation for 6 h (*n* = 5). (H) Real‐time PCR analysis of the mRNA levels of *VCAM‐1* and *ICAM‐1* in *MCT1* KD or control HUVECs stimulated with 10 mM lactate or vehicle for 3 h (*n* = 6). Data were expressed as mean ± SEM. Statistical significance was assessed using an unpaired, two‐tailed Student's *t*‐test (B and C) or one‐way ANOVA with Tukey's post hoc test (E, G, H). Compared with the without plaque group, **p < 0.01 (B). Compared with the WT group, **p < 0.01 (C). The composition of the two groups delineated by horizontal lines, *p < 0.05 and **p < 0.01 (E, G, H).

We next investigated whether ECs can take up extracellular lactate through monocarboxylic acid transporters (MCTs) to regulate VCAM‐1 and ICAM‐1 expression. Among all the transporters, MCT1 was prominently expressed in HUVECs, and its level was significantly increased by lactate treatment (Figure 2A,B). Minimal MCT1 expression was observed in the intima of human carotid arteries without plaques. However, a significant increase in MCT1 expression levels was observed in the intima of human atherosclerotic carotid arteries (Figure 2A,B). Using a publicly available single‐cell RNA‐sequencing (scRNA‐seq) dataset (GSE150089) from mouse atherosclerotic aortas, we found that Mct1 was the primary isoform in the EC cluster. In contrast, Mct2, Mct3, and Mct4 were expressed to a lesser extent, whereas Slc5a8 and Slc5a12 were not detectable in the EC cluster (Figure S2C). Additionally, *en face* staining and real time‐PCR confirmed the upregulation of Mct1 in aortic ECs from *Apoe* ^−/−^ mice (Figures 2C and S2D). Previous studies have shown that MCT4 plays a major role in the export of lactate,^15^ whereas MCT1 has been reciprocally characterized to function in the intracellular influx of lactate to ECs in many inflammatory diseases.^9^, ^10^, ^13^ Thus, we hypothesized that MCT1 could mediate lactate influx‐induced endothelial inflammation in the setting of atherosclerosis. To this end, we used the MCT1‐specific inhibitor AZD3965, which is currently in clinical trials (ClinicalTrials.gov identifier: NCT01791595), and found that MCT1 inhibition suppressed lactate‐induced VCAM‐1 and ICAM‐1 expression in HUVECs (Figure 2D,E). Similarly, knockdown (KD) of *MCT1* via siRNA against human *MCT1* significantly decreased VCAM‐1 and ICAM‐1 protein and mRNA levels in HUVECs (Figure 2F–H). Accordingly, the in vitro leukocyte‒endothelium interaction assay revealed that the lactate‐induced increase in monocyte adherence to ECs was significantly compromised by *MCT1* KD (Figure S2E).

### 2.3 Generation of endothelial‐targeting nanoparticles containing siRNAs against the mouse *Mct1* gene

To achieve in vivo KD of MCT1 in ECs, we generated endothelial‐targeting nanoparticles containing a siRNA against the mouse *Mct1* gene (si*Mct1*). PEG–PEI is a copolymer composed of cationic polyethylene imine (PEI) and polyethylene glycol (PEG). It has been extensively studied and has been shown to significantly improve the delivery of siRNA both in vitro and in vivo.^16^, ^17^, ^18^ In this study, we prepared functionalized DSPE–PEG–PEI/Pep/siRNA nanoparticles, which display a targeting peptide (Pep, VHPKQHR) against VCAM‐1 expressed on inflamed endothelium and simultaneously encapsulate control or Mct1 siRNA (si*ctl* or si*Mct1*) in their core (Figure S3A). Briefly, we synthesized DSPE–PEG–PEI, DSPE–PEG–Pep, DSPE–PEG–PEI/Pep, and DSPE–PEG–PEI/Pep/siRNA complexes in succession. To characterize these targeted nanoparticles further, ^1^H nuclear magnetic resonance (NMR) spectroscopy was used to determine the structures of DSPE–PEG–PEI and DSPE–PEG–Pep. Proton signals corresponding to PEI were observed in the range of 2.9–2.3 ppm, whereas signals corresponding to the CVHPKQHR peptide segment appeared at 7.5–7.1 ppm, which is consistent with the structure of benzene (Figure S3B). Additionally, FTIR spectra were used to confirm the compositions of DSPE–PEG–PEI, DSPE–PEG–Pep, and the DSPE–PEG–PEI/Pep complex (Figure S3C). The results revealed that the peak near 1100 cm^−1^ corresponded to the stretching vibration of C‐O in the PEG chain segment, whereas the strong absorption peak at 3412 cm^−1^ was attributed to the stretching vibration of amino groups on the PEI chain segment. These data indicate the successful synthesis of the new compounds.

The ability to bind siRNA is a fundamental requirement for delivery vectors. To investigate the siRNA affinity of the vectors, an agarose gel retardation assay was performed. As shown in Figure S3D, DSPE–PEG–PEI completely retarded siRNA migration at an N/P ratio of 6, whereas this effect occurred at an N/P ratio of 4 for DSPE–PEG–PEI/Pep. This may be because, after peptide modification, the cationic segment was condensed into a shell surrounding the hydrophobic core, resulting in a compact structure with a high charge density that strengthened the electrostatic interaction with the siRNA. The morphology of DSPE–PEG–PEI/Pep/siRNA was examined via transmission electron microscopy, revealing the formation of compact nanoparticles with a size of approximately 100 nm (Figure S3E). The hydrodynamic diameter and surface potential of the DSPE–PEG–PEI/Pep/siRNA nanoparticles were determined via dynamic light scattering, which revealed that the particle size gradually decreased with an increasing N/P ratio and eventually stabilized at approximately 200 nm (Figure S3F). The zeta potential exhibited charge reversal with the increasing N/P ratio and eventually stabilized above 10 mV (Figure S3G). Positively charged nanoparticles with diameters less than 200 nm have been reported to be suitable for efficient cellular entry and internalization.

The encapsulation efficiency (EE) of the siRNAs in DSPE–PEG–PEI and DSPE–PEG–PEI/Pep was estimated by measuring the amount of encapsulated siRNA. Consistent with the results of the agarose gel experiments, when the N/P ratio was greater than 6, the siRNA EE of all the vectors was very high (≥95%), indicating that both DSPE–PEG–PEI and DSPE–PEG–PEI/Pep had strong siRNA encapsulation capabilities (Table S3). Serum stability assays of the siRNA and DSPE–PEG–PEI/Pep/siRNA nanoparticles were performed in 50% aqueous serum via agarose gel electrophoresis. All samples were incubated with an equal volume of serum at 37°C, and aliquots were obtained at fixed time points to visualize intact siRNA. Figure S3H shows the results of siRNA degradation in the serum, along with the control groups containing intact siRNA. As expected, the bands corresponding to naked siRNA became fainter after 6 h. Only a faint band appeared after 24 h, and the bands were completely degraded after 48 h, indicating that the siRNA was sensitive to degradation by serum. In contrast, the siRNA encapsulated in DSPE–PEG–PEI/Pep/siRNA nanoparticles was well protected in the serum and remained relatively intact even after 48 h. Furthermore, the in vitro cytotoxicity of DSPE–PEG–PEI/siRNA and DSPE–PEG–PEI/Pep/siRNA at different N/P ratios was evaluated in HUVECs. As shown in Figure S3I, the viability of cells exposed to both DSPE–PEG–PEI/siRNA and DSPE–PEG–PEI/Pep/siRNA remained high (>80%) at N/P ratios of up to 15. Toxicity gradually increased as the N/P ratio increased, likely due to the high density of the amino groups. On the basis of these results, an N/P ratio of 15 was selected for subsequent experiments.

### 2.4 KD of endothelial *Mct1* via siRNA‐loaded, endothelial‐targeting nanoparticles alleviates endothelial inflammation in *Apoe^−/−^* mice

**FIGURE 3:**
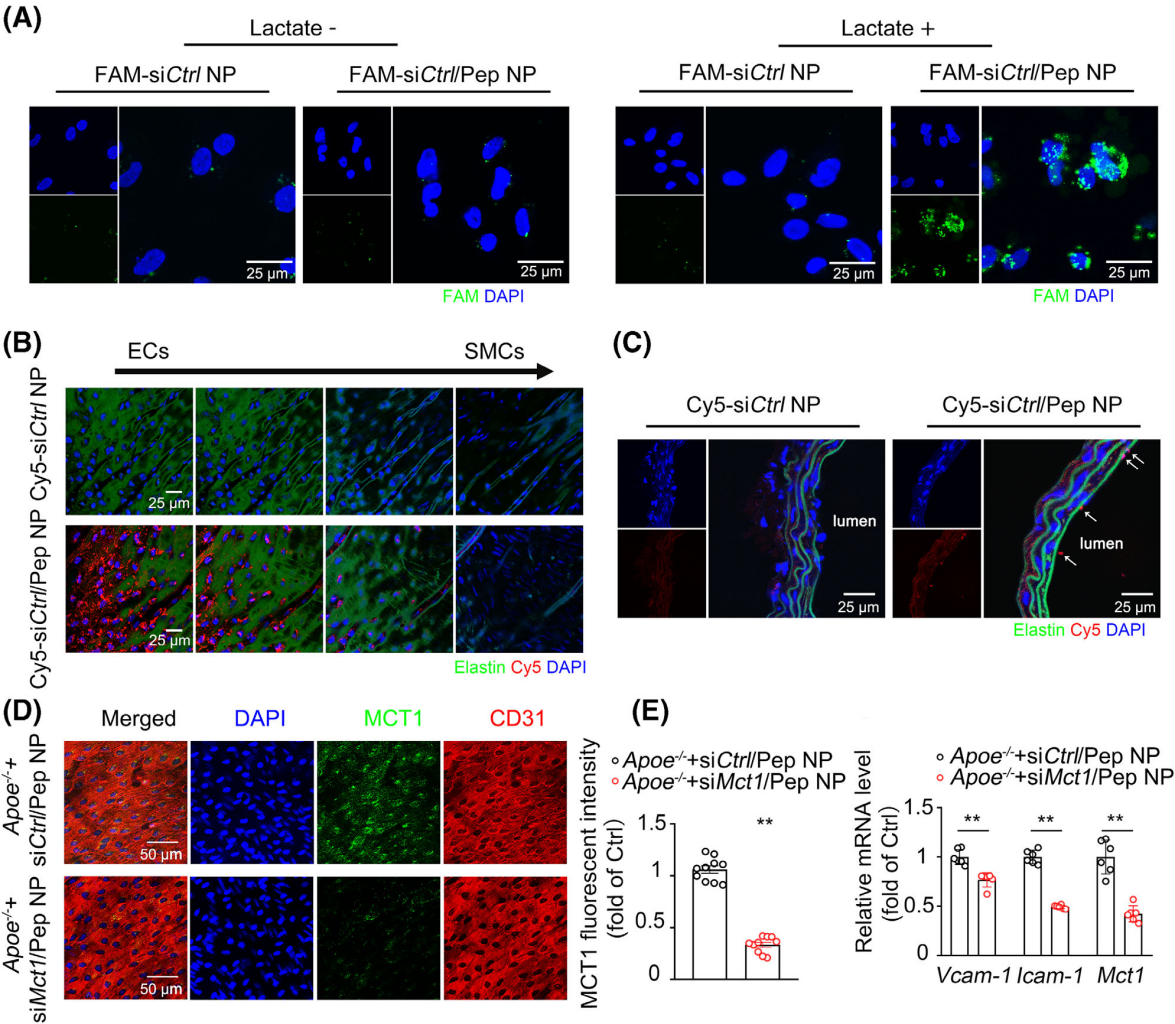
Endothelial‐specific targeting ability of DSPE–PEG–PEI/Pep nanoparticles. (A) Representative confocal image of the intracellular 5‐FAM fluorescence in HUVECs treated with FAM‐labeled DSPE–PEG–PEI/Pep (FAM‐si*Ctrl* NP) and DSPE–PEG–PEI/Pep (FAM‐si*Ctrl*/Pep NP) nanoparticles. HUVECs were treated with vehicle or 10 mM lactate for 6 h, and then subjected to FAM‐labeled DSPE–PEG–PEI/Pep (FAM‐siCtrl NP) and DSPE–PEG–PEI/Pep (FAM‐siCtrl/Pep NP) nanoparticles treatment for 3 h. (B and C) Representative confocal image of intracellular Cy5 fluorescence in endothelium layer from *Apoe* ^−/−^ mice treated with Cy5‐labeled DSPE–PEG–PEI/Pep (Cy5‐si*Ctrl* NP) and DSPE–PEG–PEI/Pep (Cy5‐si*Ctrl*/Pep NP) nanoparticles. (D) *En face* IF staining and quantification of Mct1 (green) in the aortic endothelium of 3‐month HFD‐fed *Apoe* ^−/−^ mice treated with si*Ctrl*/Pep NP, si*Mct1*/Pep NP. The endothelium was visualized by CD31 staining (red). Nuclei were counterstained with DAPI (blue) (*n* = 10 mice per group). For all bar graphs, data are the mean ± SEM, ****p < 0.0001 (unpaired, two‐tailed Student's *t*‐test). (E) Real‐time PCR analysis of the mRNA levels of *Vcam‐1* and *Icam‐1* genes in endothelial RNA obtained from *Apoe^−/−^* mice treated with DSPE–PEG–PEI/Pep/si*ctl* (si*ctl/*Pep NP) and DSPE–PEG–PEI/Pep/si*Mct1* (si*Mct1/*Pep NP) nanoparticles. Initiating biweekly treatments with either si*ctl*/Pep NP or si*Mct1*/Pep NP at 6 weeks of age, the *Apoe* ^−/−^ mice were exposed to a WD from the 8th week, and by their 14th week, endothelial RNA was extracted from mouse aortas through flushing with TRIzol reagent (*n* = 6 mice per group). For all bar graphs, data are the mean ± SEM. Statistical significance was assessed using an unpaired, two‐tailed Student's *t*‐test (D and E). Compared with the *Apoe* ^−/−^+si*Ctrl*/Pep NP group, **p < 0.01 (D and E).

To demonstrate the endothelial‐specific targeting ability of DSPE–PEG–PEI/Pep nanoparticles, we performed in vitro experiments in which DSPE–PEG–PEI/Pep/si*ctl*‐FAM nanoparticles loaded with 5‐FAM‐labeled si*ctl* were coincubated with 10 mM lactate or vehicle‐treated HUVECs. By observing the intracellular fluorescence, we found that lactate‐treated ECs specifically took up DSPE–PEG–PEI/Pep/si*ctl*‐FAM nanoparticles (Figure 3A). For in vivo experiments, we administered DSPE–PEG–PEI/Pep/si*ctl*‐Cy5 nanoparticles loaded with Cy5‐labeled si*ctl* to *Apoe* ^−/−^ mice fed a WD for 1 month via tail vein injection and observed that the DSPE–PEG–PEI/Pep/si*ctl*‐Cy5 nanoparticles were specifically taken up by ECs (Figure 3B,C).

To determine whether Mct1 inhibition could alleviate endothelial inflammation in atherosclerosis, we administered DSPE–PEG–PEI/Pep/si*ctl* and DSPE–PEG–PEI/Pep/si*Mct1* nanoparticles to *Apoe^−/−^* mice fed a WD for 1 month by tail vein injection. si*Mct1*‐containing nanoparticles significantly decreased Mct1 levels in the aortic endothelium (Figure 3D) and reduced VCAM‐1 and ICAM‐1 expression in the aortic ECs of *Apoe* ^−/−^ mice (Figure 3E). These data suggest that Mct1 facilitates lactate uptake into ECs, thereby promoting vascular inflammation.

### 2.5 NADH production due to lactate oxidation is critical in endothelial inflammation

**FIGURE 4:**
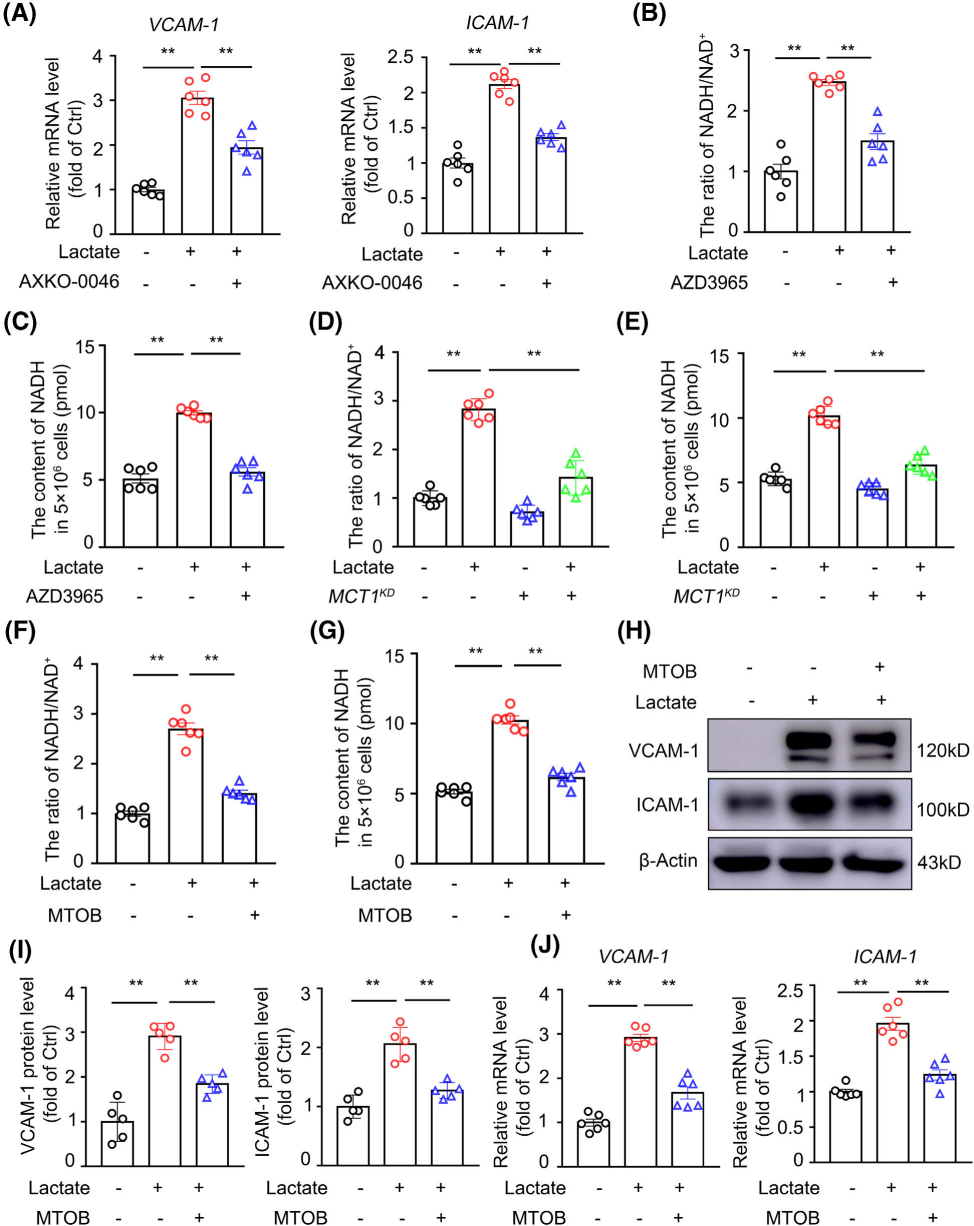
The production of NADH due to lactate oxidation are critical in endothelial inflammation. (A) Real‐time PCR analysis of the mRNA levels of *VCAM‐1* and *ICAM‐1* genes in HUVECs pretreated with the LDH‐B selective inhibitor AXKO‐0046 dihydrochloride (50 nM) for 30 min and then stimulated with lactate (10 mM) or vehicle for 3 h (*n* = 6). (B and C) The ratio of NADH/NAD^+^ (B) and the content of NADH (C) measured using a NADH/NAD^+^ Quantitation Kit in lactate (10 mM for 3 h)‐treated HUVECs pretreated with the MCT1 inhibitor AZD3965 (500 nM) for 24 h (*n* = 6). (D and E) The ratio of NADH/NAD^+^ (D) and the content of NADH (E) in lactate‐treated MCT1 KD or control HUVECs (*n* = 6). (F and G) The ratio of NADH/NAD^+^ (F) and the content of NADH (G) in lactate‐treated HUVECs pretreated with the NADH depletor MTOB (2.5 mM) for 24 h (*n* = 6). (H and I) Western blot detection and quantification of the protein levels of VCAM‐1 and ICAM‐1 in HUVECs pretreated with the NADH depletor MTOB (2.5 mM) for 24 h and then stimulated with 10 mM lactate or vehicle for 6 h (*n* = 5). (J) Real‐time PCR analysis of the mRNA levels of *VCAM‐1* and *ICAM‐1* genes in HUVECs pretreated with the NADH depletor MTOB (2.5 mM) for 24 h and then stimulated with 10 mM lactate or vehicle for 3 h (*n* = 6). For all bar graphs, data are the mean ± SEM. The composition of the two groups delineated by horizontal lines, **p < 0.01; one‐way ANOVA with Tukey's post hoc test (A, B, C, D, E, F, G, I, J).

Lactate can serve as an energy substrate through its oxidation to pyruvate, a reaction catalyzed by LDH‐B and coupled to NADH production (Figure S4A). Using the LDH‐B selective inhibitor AXKO‐0046 dihydrochloride, we found that LDH‐B inhibition significantly decreased lactate‐induced VCAM‐1 and ICAM‐1 expression in ECs (Figure 4A), indicating that the conversion to pyruvate mediates lactate‐induced endothelial inflammation. To investigate whether pyruvate itself has proinflammatory effects, HUVECs were treated with vehicle or 10 mM pyruvate for 0 to 24 h. In contrast to lactate, pyruvate administration did not increase VCAM‐1 or ICAM‐1 expression in ECs (Figure S4B,C).

Our observations of inconsistent effects between lactate and pyruvate suggest that NADH, generated through the oxidation of lactate to pyruvate by LDH‐B, may play a role in the proinflammatory effect of lactate. To test this possibility, we assayed intracellular NADH and NAD^+^ levels and detected significant increases in the NADH content and the NADH/NAD^+^ ratio in lactate‐treated ECs, effects that were compromised by pharmacological or genetic inhibition of MCT1 (Figures 4B–E and S4D,E). To examine the functional relationship between an increased NADH/NAD^+^ ratio and endothelial inflammation, 4‐methylthio‐2‐oxobutyric acid (MTOB) was used to pharmacologically deplete the NADH content (Figures 4F,G and S4F). Pretreatment with MTOB significantly attenuated lactate‐induced VCAM‐1 and ICAM‐1 expression at both the protein and mRNA levels in ECs (Figure 4H–J). These data indicate that the oxidation of lactate to pyruvate, accompanied by the concomitant production of NADH, is necessary for the proinflammatory effects of lactate.

### 2.6 CtBP oligomerization is critical for lactate‐induced endothelial inflammation

**FIGURE 5:**
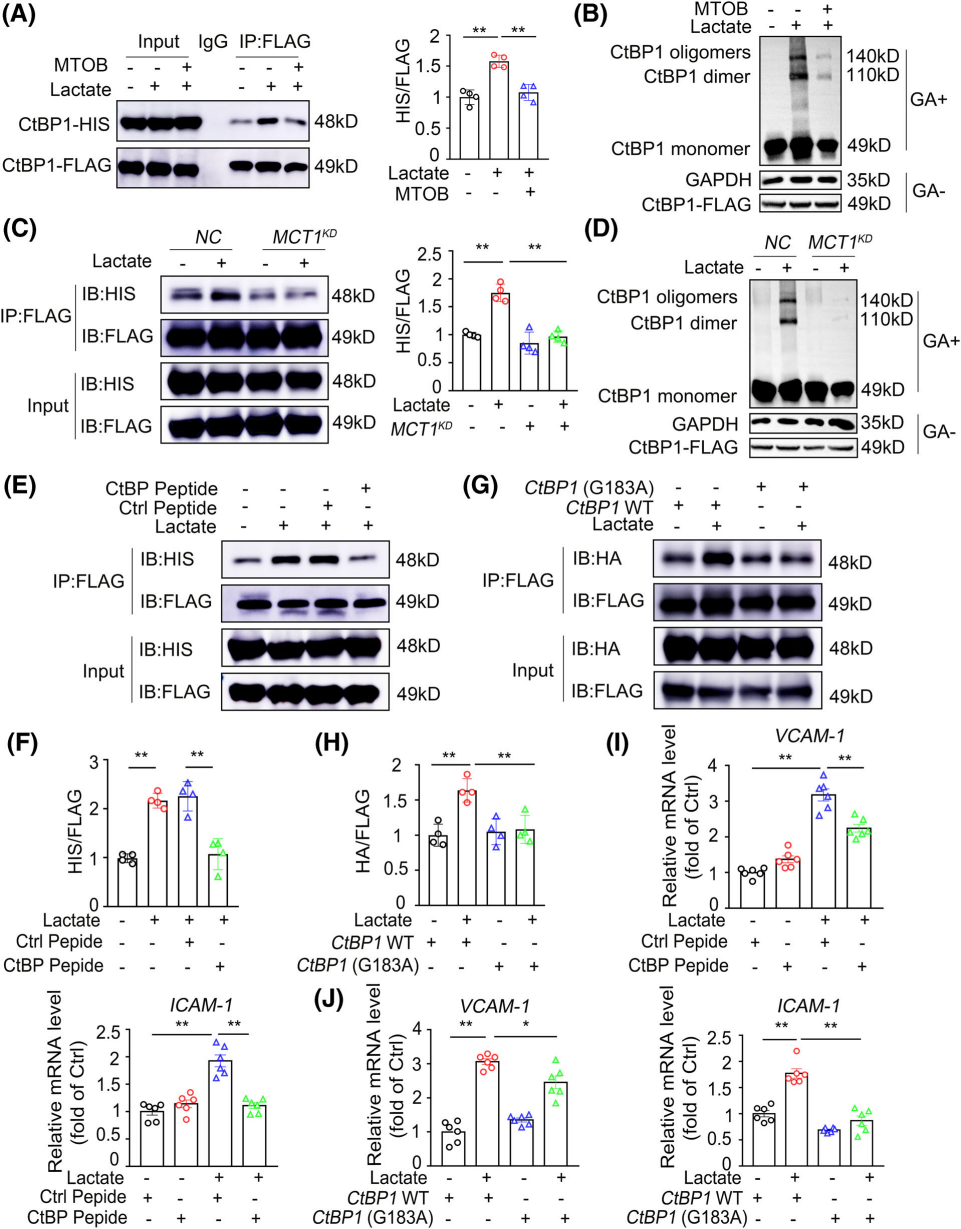
Oligomerization of CtBP1 is critical for lactate‐induced endothelial inflammation. (A) Co‐IP of CtBP1–FLAG and CtBP1–HIS in FLAG/HIS‐tagged CtBP1‐overexpressed HUVECs pretreated with the NADH depletor MTOB (2.5 mM) for 24 h and then treated with vehicle or 10 mM lactate for 3 h. Two hundred micrograms of protein lysates was precipitated with rabbit anti‐FLAG antibody followed by immunoblotting with mouse anti‐HIS antibody (*n* = 4). (B) Change of CtBP1 oligomer states in FLAG‐tagged CtBP1‐overexpressed HUVECs pretreated with the NADH depletor MTOB (2.5 mM) for 24 h and then treated with vehicle or 10 mM lactate for 3 h. The whole‐cell lysates were treated with 0.25‰ glutaraldehyde (GA) for 5 min and then analyzed by Western blot with the Flag antibody (*n* = 3). (C) Co‐IP of CtBP1–FLAG and CtBP1–HIS in FLAG/HIS‐tagged CtBP1‐overexpressed HUVECs transfected with control or si*MCT1* for 48 h and then challenged with vehicle or 10 mM lactate for 3 h (*n* = 4). (D) Change of CtBP1 oligomer states in FLAG‐tagged CtBP1‐overexpressed HUVECs transfected with control or si*MCT1* for 48 h and then challenged with vehicle or 10 mM lactate for 3 h. The whole‐cell lysates were treated with 0.25‰ glutaraldehyde (GA) for 5 min and then analyzed by Western blot with the Flag antibody (*n* = 3). (E and F) Co‐IP of CtBP1–FLAG and CtBP1–HIS in FLAG/HIS‐tagged CtBP1‐overexpressed HUVECs pretreated with Ctrl or CtBP peptide (50 µM) for 30 min and then stimulated with lactate (10 mM) or vehicle for 3 h (*n* = 4). (G and H) Co‐IP of CtBP1–FLAG and CtBP1–HA in FLAG/HA‐tagged WT CtBP1 or FLAG/HA‐tagged G183A CtBP1‐overexpressed HUVECs stimulated with lactate (10 mM) or vehicle for 3 h (*n* = 4). (I) Real‐time PCR analysis of the mRNA levels of *VCAM‐1* and *ICAM‐1* genes in HUVECs pretreated with Control or CtBP peptide (20 µM) for 30 min and then stimulated with lactate (10 mM) or vehicle for 3 h (*n* = 6). (J) Real‐time PCR analysis of the mRNA levels of *VCAM‐1* and *ICAM‐1* genes in FLAG/HA‐tagged WT CtBP1 or FLAG/HA‐tagged G183A CtBP1‐overexpressed HUVECs stimulated with lactate (10 mM) or vehicle for 3 h (*n* = 6). For all bar graphs, data are the mean ± SEM. The composition of the two groups delineated by horizontal lines, *p < 0.05 and **p < 0.01; one‐way ANOVA with Tukey's post hoc test (A, C, F, H, I, J).

NADH is an allosteric regulator of C‐terminal binding proteins (CtBPs), which function as transcriptional corepressors to affect gene transcription.^19^, ^20^, ^21^ Given that lactate increases *VCAM‐1* and *ICAM‐1* mRNA levels, we evaluated whether CtBP plays a role in lactate‐induced endothelial inflammation. Vertebrates possess two CtBP proteins, CtBP1 and CtBP2, which have similar functions.^22^, ^23^ Although both proteins were expressed at similar levels in resting ECs, only CtBP1 was significantly upregulated when these cells were treated with lactate (Figure S5A). As a result, our study focused on the role of CtBP1. NADH recruitment induces CtBP dimerization or higher‐order oligomerization, linking enzymatic function with transcriptional activity.^20^, ^24^ To examine CtBP oligomerization, we coexpressed HA‐tagged and FLAG‐tagged CtBP1 in lactate‐treated ECs and performed immunoprecipitation (IP) with an anti‐FLAG antibody. Lactate administration greatly increased the amount of CtBP1–HA associated with CtBP1–FLAG (Figure S5B). In addition, using glutaraldehyde cross‐linking studies, we observed that lactate treatment indeed increased the levels of CtBP1 dimers and higher‐order oligomers (Figure S5C). As expected, NADH depletion (Figure 5A,B) or MCT1 KD (Figure 5C,D), which decreased lactate‐induced endothelial inflammation, also disrupted CtBP1 oligomerization. These results indicate that interfering with CtBP oligomerization might negate the proinflammatory effect of lactate. To further explore the causal relationship between CtBP oligomerization and lactate‐induced endothelial inflammation, we introduced a synthetic peptide (Figure 5E), which has been shown to block CtBP oligomerization,^20^ or overexpressed an NADH‐insensitive mutant of CtBP1 (G183A).^25^ Both treatments significantly decreased lactate‐induced CtBP1 oligomerization in ECs (Figure 5E–H) and eradicated lactate‐induced VCAM‐1 and ICAM‐1 expression at both the mRNA (Figure 5I,J) and protein (Figure S5D,E) levels. Together, these data suggest that CtBP oligomerization contributes to lactate‐induced endothelial inflammation.

### 2.7 FOXP1 binds to monomeric CtBP to transrepress the expression of adhesion molecules

**FIGURE 6:**
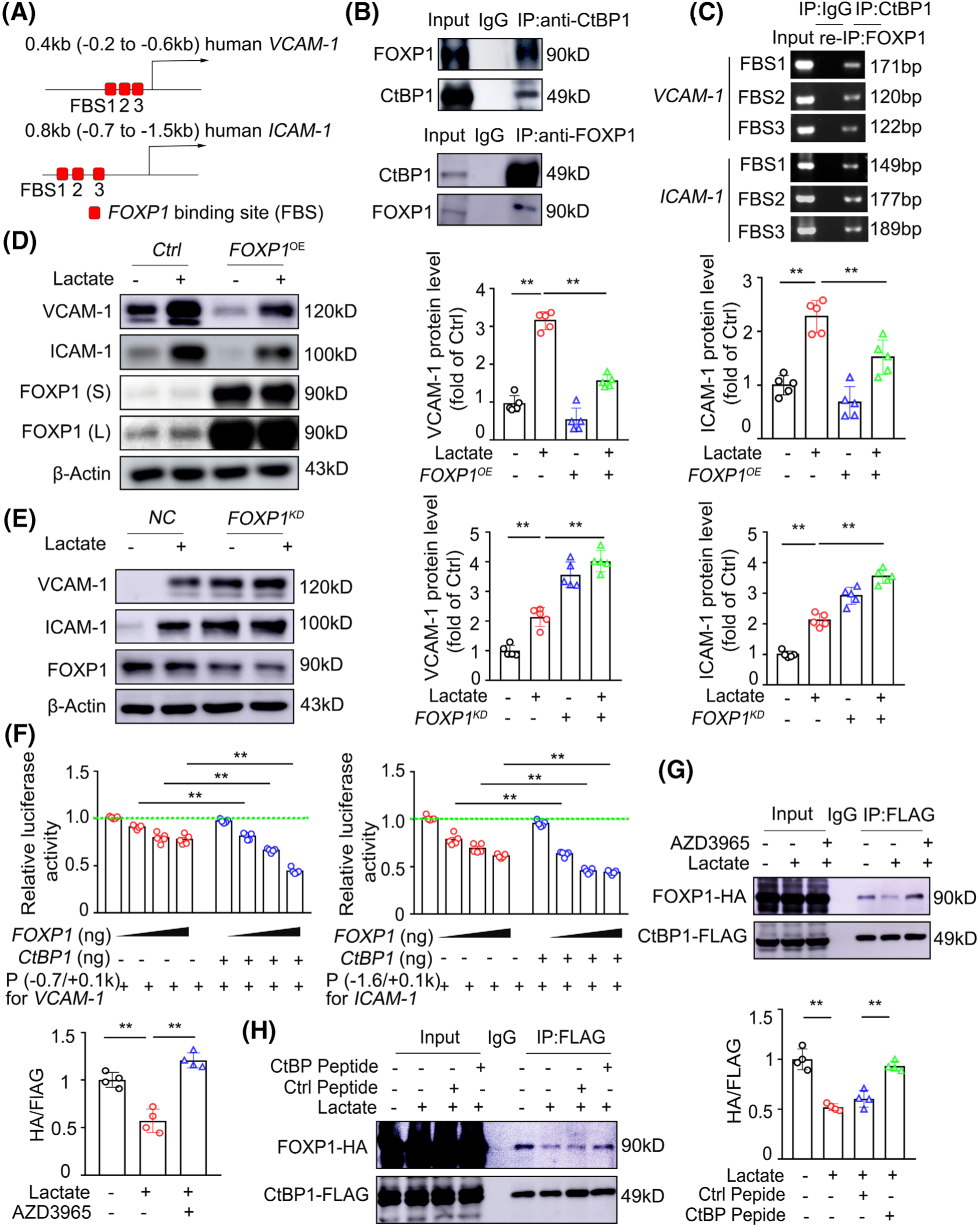
FOXP1 binds with monomeric CtBP to transrepress the expression of adhesion molecules. (A) Sequence analysis showing FOXP1 binding sites (red square) in the proximal promoter of *VCAM‐1* and *ICAM‐1* genes. (B) Co‐IP of endogenous CtBP1 and FOXP1 in HUVECs. Upper: IP was performed with mouse anti‐CtBP1 followed by immunoblotting with rabbit anti‐FOXP1 and mouse anti‐CtBP1. Lower: IP was performed with rabbit anti‐FOXP1 followed by immunoblotting with mouse anti‐CtBP1 and rabbit anti‐FOXP1 (*n* = 4). (C) Sequential chromatin immunoprecipitation (SeqChIP; also referred to as Re‐ChIP) assay showing the accumulation of the complex of FOXP1 and CtBP1 on the promoter of *VCAM‐1* and *ICAM‐1* genes in HUVECs (*n* = 4). (D) Western blot detection and quantification of the protein levels of VCAM‐1 and ICAM‐1 in FOXP1‐overexpressed or control HUVECs stimulated with 10 mM lactate or vehicle for 6 h (*n* = 5). S, short exposure; L, long exposure. (E) Western blot detection and quantification of the protein levels of VCAM‐1 and ICAM‐1 in *FOXP1* KD or control HUVECs stimulated with 10 mM lactate or vehicle for 6 h (*n* = 5). (F) Luciferase reporter assay showing the activity of promoter regions of *VCAM‐1* and *ICAM‐1* genes in FOXP1 and CtBP1‐overexpressed HEK293T cells (*n* = 5). (G) Co‐IP of CtBP1–FLAG and FOXP1–HA in FLAG‐tagged CtBP1 and HA‐tagged FOXP1‐overexpressed HUVECs pretreated with the MCT1 inhibitor AZD3965 (500 nM) for 24 h followed by 10 mM lactate stimulation for 3 h (*n* = 4). (H) Co‐IP of CtBP1–FLAG and FOXP1–HA in FLAG‐tagged CtBP1 and HA‐tagged FOXP1‐overexpressed HUVECs pretreated with control or CtBP peptide (50 µM) for 30 min and then stimulated with lactate (10 mM) or vehicle for 3 h (*n* = 4). For all bar graphs, data are the mean ± SEM. Statistical significance was assessed using one‐way ANOVA (D, E, G, H) or two‐way ANOVA (F) with Tukey's post hoc test. The composition of the two groups delineated by horizontal lines, **p < 0.01 (D, E, F, G, H).

CtBP1 plays a key role in gene transcription by binding to various transcriptional repressors.^26^, ^27^, ^28^ Using the STRING and HitPredict databases, which assess protein‒protein interactions, we predicted potential transcription factors that might associate with CtBP1 to regulate the expression of adhesion molecules and found that eleven targets (FOXP1, ZEB1, HDAC1, KDM1A, ZEB2, CBX4, TGIF1, IKZF1, APC, AN217, and MECOM) were present in both databases (Figure S6A). Using the JASPAR database and setting the relative profile score threshold at 85%, we found that the promoter regions of both the *VCAM‐1* and *ICAM‐1* genes contained binding elements exclusively for FOXP1 and ZEB1 (Figure 6A). In light of studies showing that FOXP1 can transcriptionally repress the NLRP3 inflammasome and inhibit the development of atherosclerosis,^29^ we hypothesized that CtBP1 might interact with FOXP1 to modulate *VCAM‐1* and *ICAM‐1* gene transcription. Consistent with this notion, we observed a direct association between FOXP1 and CtBP1, as well as the accumulation of this complex in the promoters of the *VCAM‐1* and *ICAM‐1* genes in quiescent ECs (Figure 6B,C). Furthermore, lactate reduced FOXP1 accumulation at these promoters, but this reduction was disrupted by treatment with the MCT1 inhibitor AZD3965 (Figure S6B,C). To investigate the functional role of FOXP1 in endothelial inflammation, we overexpressed or silenced FOXP1 in ECs. We found that FOXP1 overexpression suppressed (Figures 6D and S6D). In contrast, FOXP1 KD potentiated (Figures 6E and S6E) and lactate induced *VCAM‐1* and *ICAM‐1* expression at both the mRNA and protein levels. Using a luciferase reporter assay, we also observed that FOXP1 overexpression dose‐dependently inhibited the promoter activity of the *VCAM‐1* and *ICAM‐1* genes in HEK293T cells and that CtBP1 overexpression markedly enhanced these effects (Figures 6F and S6F). However, mutation of the putative FOXP1 binding sites (FBSs) resulted in the loss of responsiveness of the *VCAM‐1* and *ICAM‐1* promoters to FOXP1 and CtBP1 overexpression (Figure S6F,G). These results indicate that CtBP1 increases the transrepressive effect of FOXP1 on the *VCAM‐1* and *ICAM‐1* promoters.

Given that lactate treatment induces CtBP1 oligomerization, we examined how the oligomerization status of CtBP1 affects its ability to bind to FOXP1 and its transrepressive activity. We coexpressed FOXP1–HA‐ and CtBP1–FLAG‐tagged constructs in ECs and performed IP with an anti‐FLAG antibody. Lactate treatment significantly decreased the association between FOXP1 and CtBP1, an effect disrupted by the MCT1 inhibitor AZD3965 or the CtBP peptide (Figure 6G,H). These data demonstrate that the monomeric, but not dimeric, form of CtBP associates with FOXP1 to transrepress adhesion molecule expression and that lactate/NADH‐dependent CtBP oligomerization prevents its binding to FOXP1, at least partially alleviating FOXP1's transrepressive activity.

### 2.8 Endothelial‐specific *Mct1* silencing reduces atherogenesis in a FOXP1‐dependent manner

**FIGURE 7:**
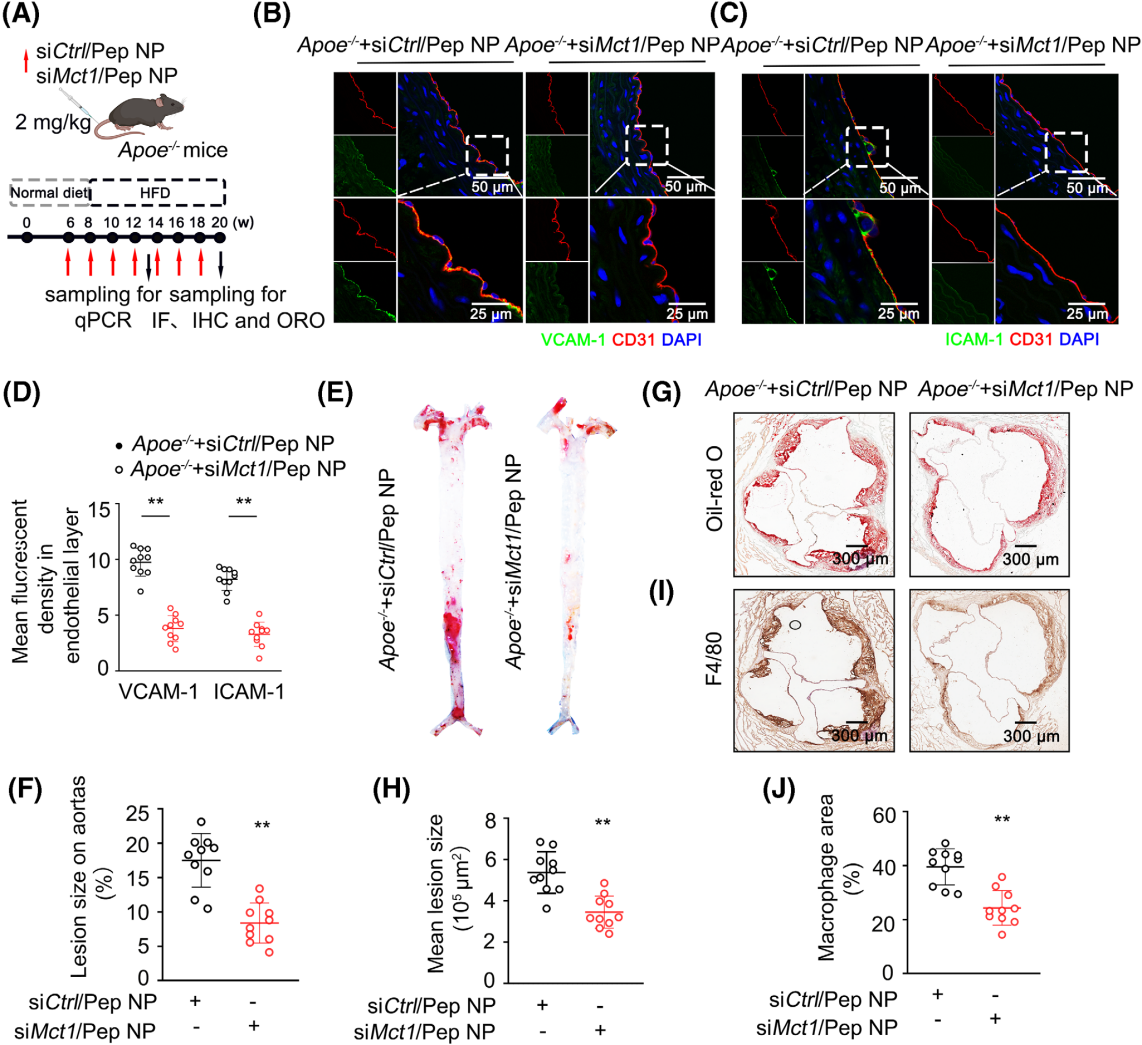
Nanoparticle‐mediated endothelial‐specific MCT1 silencing reduces atherogenesis. (A) Schematic diagram showing the experimental design for treatment of *Apoe^−/−^* mice with DSPE–PEG–PEI/Pep/si*ctl* (si*Ctrl/*Pep NP) and DSPE–PEG–PEI/Pep/si*Mct1* (si*Mct1/*Pep NP) nanoparticles. (B–D) IF staining and quantification of ICAM‐1 and VCAM‐1 in the aortic endothelium of 3‐month HFD‐fed *Apoe* ^−/−^ mice treated with si*Ctrl/*Pep NP and si*Mct1/*Pep NP. The endothelium was visualized by CD31 staining (red). Nuclei were counterstained with DAPI (blue) (*n* = 10 mice per group). (E and F) Oil Red O‐stained *en face* aortic preparations from 3‐month HFD‐fed *Apoe* ^−/−^ mice injected with si*Ctrl/*Pep NP and si*Mct1/*Pep NP (E), and quantification of the Oil Red O‐stained areas (F) (*n* = 10 mice per group). (G and H) Oil Red O staining of aortic roots of the 3‐month HFD‐fed *Apoe* ^−/−^ mice injected with si*Ctrl/*Pep NP and si*Mct1/*Pep NP (G) and quantification of the Oil Red O‐stained areas (H) (*n* = 10 mice per group). (I and J) IHC staining of F4/80 (I) and quantification of F4/80^+^ areas (J) in aortic roots of 3‐month‐WD‐fed *Apoe* ^−/−^ mice injected with si*Ctrl/*Pep NP and si*Mct1/*Pep NP (*n* = 10 mice per group). For all bar graphs, data are the mean ± SEM. Statistical significance was assessed using an unpaired, two‐tailed Student's *t*‐test (D, F, H, J). Compared with the *Apoe* ^−/−^+si*Ctrl*/Pep NP group, **p < 0.01 (D, F, H, J).

Considering that MCT1 aids in the uptake of lactate into ECs for vascular inflammation, we aimed to ascertain whether silencing Mct1 in vivo ameliorates atherosclerosis. Although MCT1 inhibition via small molecules is currently under investigation in cancer clinical trials, the systemic delivery of such inhibitors may lead to off‐target toxicity. To achieve EC‐specific KD of *Mct1*, immediately after initiating a WD to induce atherosclerosis, *Apoe* ^−/−^ mice were administered DSPE–PEG–PEI/Pep/si*ctl* and DSPE–PEG–PEI/Pep/si*Mct1* nanoparticles via tail vein injection at biweekly intervals for a total duration of 14 weeks (Figure 7A). We noted that endothelial Mct1 deficiency had no effect on plasma cholesterol levels either before (168.3 vs. 159.3 mg/dL for the si*ctl* group and si*Mct1* group) or after (789.6 vs. 804.8 mg/dL for the si*ctl* group and si*Mct1* group) the introduction of the WD. However, we observed a significant decrease in VCAM‐1 and ICAM‐1 levels in the aortic endothelium of *Apoe* ^−/−^ mice treated with DSPE–PEG–PEI/Pep/si*Mct1* nanoparticles (Figure 7B–D). The suppression of *Mct1* expression in *Apoe* ^−/−^ mice resulted in robust prevention of atherosclerosis, as indicated by Oil Red O staining of whole aortas (Figure 7E,F) and the aortic sinus (Figure 7G,H). Immunohistochemical staining for F4/80 revealed a decrease in the number of macrophages in the aortic sinus of *Apoe* ^−/−^ mice treated with DSPE–PEG–PEI/Pep/si*Mct1* nanoparticles (Figure 7I,J).

**FIGURE 8:**
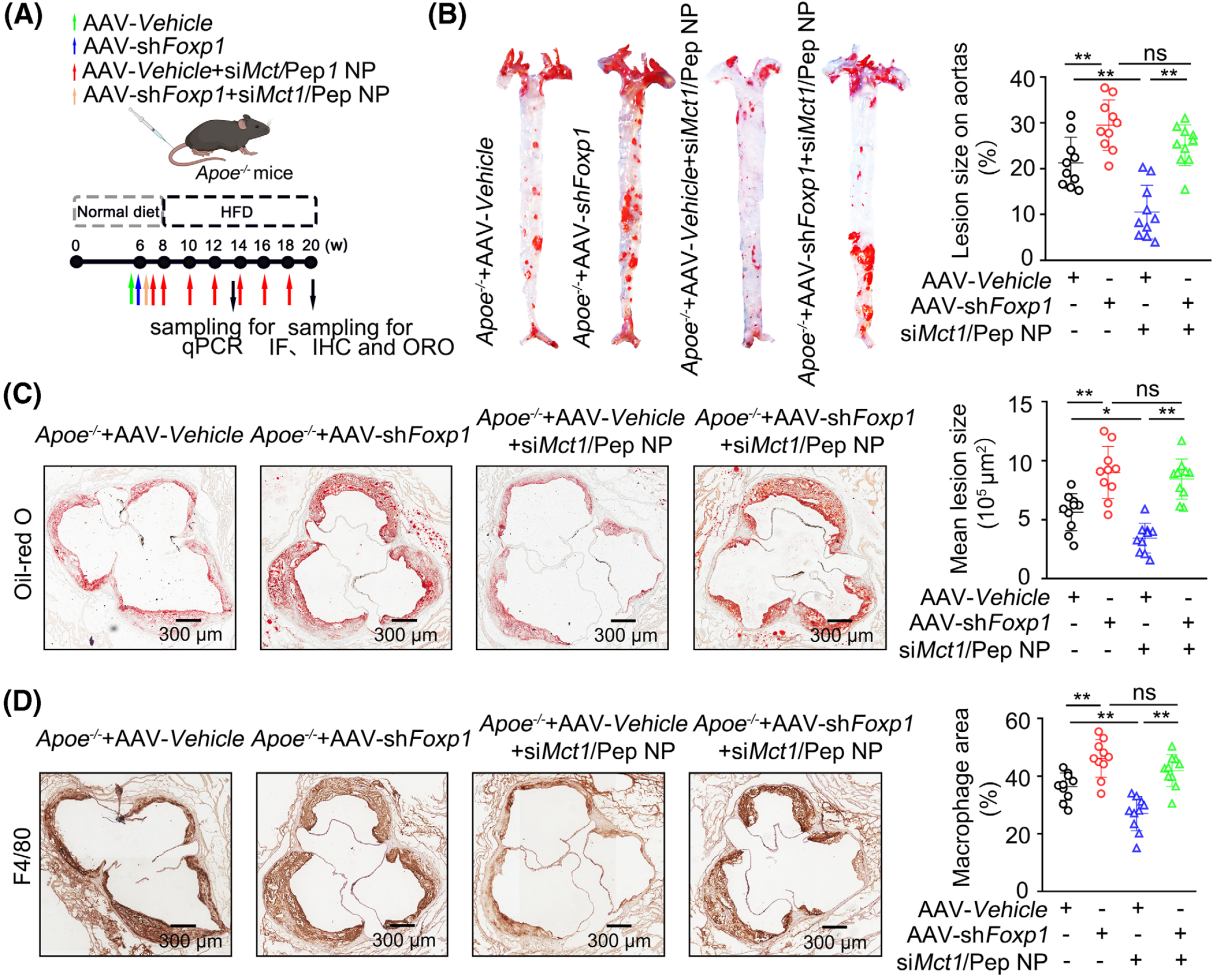
Endothelial‐specific *Mct1* silencing reduces atherogenesis in a FOXP1‐dependent manner. (A) Schematic diagram showing the experimental design for treatment of *Apoe^−/−^* mice with AAV–*Cdh5*–*Vehicle*, AAV–*Cdh5*–sh*Foxp1*, AAV–*Cdh5*–*Vehicle* plus si*Mct1*/Pep NP, and AAV–*Cdh5*–sh*Foxp1* plus si*Mct1*/Pep NP. (B) Oil Red O‐stained *en face* aortic preparations from 3‐month HFD‐fed *Apoe* ^−/−^ mice injected with AAV–*Cdh5*–*Vehicle*, AAV–*Cdh5*–sh*Foxp1*, AAV–*Cdh5*–*Vehicle* plus si*Mct1*/Pep NP, and AAV–*Cdh5*–sh*Foxp1* plus si*Mct1*/Pep NP, and quantification of the Oil Red O‐stained areas (*n* = 10 mice per group). (C) Oil Red O staining of aortic roots and quantification of the Oil Red O‐stained areas (*n* = 10 mice per group). (D) IHC staining of F4/80 and quantification of F4/80^+^ areas in aortic roots (*n* = 10 mice per group). For all bar graphs, data are the mean ± SEM. The composition of the two groups delineated by horizontal lines, *p < 0.05 and **p < 0.01; one‐way ANOVA with Tukey's post hoc test (B, C, D).

Given the robust in vitro efficacy of lactate in alleviating the transrepressive effect of FOXP1 on VCAM‐1 and ICAM‐1 expression, we aimed to ascertain whether silencing Foxp1 in vivo could obstruct the antiatherosclerotic effect of Mct1 silencing in *Apoe* ^−/−^ mice. To achieve this goal, we specifically silenced *Foxp1* in ECs from *Apoe* ^−/−^ mice via EC‐enhanced AAV9‐mediated *Foxp1* KD, which was under the control of the EC‐specific Cdh5 promoter (AAV–Cdh5–sh*Foxp1*) (Figures 8A and S7). Following gene delivery, the *Apoe* ^−/−^ mice were fed a WD and administered DSPE–PEG–PEI/Pep/si*ctl* or DSPE–PEG–PEI/Pep/si*Mct1* nanoparticles (Figure 8A). *Apoe* ^−/−^ mice that received AAV–Cdh5–sh*Foxp1* presented an increase in the atherosclerotic plaque area (Figure 8B,C). Furthermore, these mice had significantly greater macrophage contents within the plaque compared with mice injected with the vector (Figure 8D). Although treatment with *Mct1* siRNA‐conjugated nanoparticles reduced atherogenesis in *Apoe* ^−/−^ mice, it did not decrease the plaque area or macrophage content within the plaque in *Apoe* ^−/−^ mice that received AAV–Cdh5–sh*Foxp1* (Figure 8B–D). These findings demonstrate that the reduction in atherogenesis through endothelial‐specific *Mct1* silencing is dependent on FOXP1.

## 3 DISCUSSION

This study reveals a novel role for lactate in instigating vascular inflammation during atherosclerosis by coordinating the MCT1/NADH/CtBP1‐dependent transrepressive activity of FOXP1 in ECs. We found that the adhesion molecules VCAM‐1 and ICAM‐1 are direct downstream targets of FOXP1. Furthermore, monomeric CtBP1, but not its dimeric form, interacts with FOXP1 to amplify its transrepressive impact on VCAM‐1 and ICAM‐1 in resting ECs. However, the simultaneous generation of NADH, resulting from the conversion of lactate to pyruvate, triggers CtBP1 oligomerization, causing it to disengage from FOXP1 and reducing its transrepressive function. These lactate‐mediated events can occur even in the presence of glucose. Inhibiting the MCT1‐mediated entry of lactate into ECs mitigates vascular inflammation and ameliorates atherosclerosis. A schematic model depicting these processes is provided in Figure S7C.

In this study, we noted an increase in lactate levels in the blood and aortic tissues of mice with atherosclerosis. The increase in lactate due to atherosclerosis is likely attributed to multiple causes. The liver (accounting for 60%) and kidneys (accounting for 30%) are primarily responsible for the recycling and clearance of lactate from the circulatory system.^30^ Substantial clinical evidence has demonstrated significant associations between diverse liver diseases and vascular inflammation, as well as atherosclerosis.^31^, ^32^ Animal studies have also revealed that *Apoe* ^−/−^ mice fed a high‐fat WD suffer from liver damage, which is characterized by the rapid onset of hepatic steatosis, inflammation, and fibrosis.^33^, ^34^ Therefore, the hindered clearance of lactate could represent a factor involved in the increased lactate levels associated with atherosclerosis. Another possible contributing factor could be the increased production of lactate by various tissues. This is mainly because local hypoxia and inflammation can stimulate both anaerobic and aerobic glycolysis, thereby increasing local and systemic lactate levels. In the context of chronic inflammation in atherosclerosis, a variety of vascular cells in the vascular wall, including VSMCs, ECs, and macrophages, exhibit increased glycolysis levels.^11^, ^35^ This increase in glycolysis can subsequently result in increased lactate levels within local vascular tissues.

Lactate has long been viewed as a mere byproduct of cellular anaerobic glucose breakdown. However, in recent decades, elevated serum lactate levels have become a crucial biomarker in critical care, and lactate has been found to act as a signaling molecule that causes endothelial barrier dysfunction in sepsis.^13^ To explore whether the accumulation of lactate leads to vascular damage, we administered lactate through intraperitoneal injection and discovered that it exacerbated vascular inflammation in mice with atherosclerosis. However, the administration of lactate had no significant effect on serum lactate levels or vascular inflammation in WT mice, possibly because of the immediate and rapid clearance of lactate. This finding aligns with a recent study that revealed that serum lactate levels peak at 13 min and return to baseline levels 37 min after the injection of lactate into WT mice.^36^

ECs have the ability to both produce and utilize lactate. Although ECs at rest display relatively lower levels of glycolysis,^37^ inflamed ECs exhibit higher levels of glycolysis and may contribute, at least in part, to the lactate pool within the microenvironment of atherosclerotic lesions.^38^ Moreover, our current research suggests that ECs also use lactate to intensify their own functional dysregulation. ECs respond to lactate stimulation in various pathological microenvironments, including tumor tissues, fibrotic myocardial tissues, and sepsis. In these situations, hyperglycolytic ECs produce lactate but still take up extracellular lactate via MCT1, thereby promoting tumor angiogenesis and the EndoMT or impairing endothelial barrier function.^9^, ^10^, ^13^ Our current study revealed that lactate‐induced endothelial inflammation necessitates MCT1‐mediated lactate influx. Using si*Mct1*‐loaded nanoparticle conjugates, we discovered that targeting the endothelial MCT1 transporter could improve atherosclerosis. Therefore, in the context of atherosclerosis, locally accumulated lactate, including that released by hyperglycolytic ECs, may act on neighboring resting ECs, triggering their inflammatory activation. This possibility merits further comprehensive experimental investigation. Additionally, we cannot exclude the effects of intracellular lactate produced by ECs on vascular inflammation in this study.

Lactate, a key metabolic intermediate, can be transformed into pyruvate, which then participates in mitochondrial oxidative phosphorylation or initiates fatty acid synthesis (FAS) in normal, inflammatory, or tumor tissues, sometimes even surpassing glucose.^39^ Moreover, pyruvate intensifies the inflammatory response in inflammatory cells by fueling de novo FAS.^40^ On the other hand, the mitochondrial use of pyruvate has been shown to curb the inflammatory response of ECs.^41^ Our current study revealed that the administration of pyruvate did not increase VCAM‐1 or ICAM‐1 expression in ECs. This could be due to a balance between the proinflammatory effects triggered by pyruvate‐fueled FAS and the anti‐inflammatory effects resulting from mitochondrial oxidation. The conversion of lactate to pyruvate is catalyzed by LDH‐B, which simultaneously reduces NAD^+^ to NADH. Our data on ECs treated with an MCT1 inhibitor, LDH‐B inhibitor, or NADH depletor suggest that NADH production by lactate oxidation plays a role in the proinflammatory effect of lactate. An increase in NADH has also been shown to mediate the inflammatory response in LPS‐stimulated macrophages and fibroblasts in pulmonary hypertension.^20^, ^42^ In addition to its role in activating the NADH/CtBP pathway, lactate has been shown to elicit a number of biological effects, either through its interaction with the lactate receptor GRP81 or by instigating lactylation.^43^, ^44^ Whether these mechanisms are involved in lactate‐induced endothelial inflammation requires further investigation.

CtBP1, a transcriptional corepressor, undergoes oligomerization in response to elevated NADH levels.^20^ This finding implies that CtBP1 modulates gene expression in response to alterations in cellular metabolism. Our research revealed that the proinflammatory effect of lactate in ECs was suppressed by blocking MCT1, depleting NADH, overexpressing a mutant of CtBP1 that is insensitive to NADH, or using a synthetic peptide that inhibits CtBP1 oligomerization. These results indicate that the actions of lactate are dependent on CtBP1 oligomerization induced by NADH. Lactate is oxidized to pyruvate by lactate dehydrogenase, which generates NADH by reducing NAD^+^. Although we noted an increase in the NADH level in ECs treated with lactate, we did not observe a significant decrease in the NAD^+^ level. This could be attributed to the fact that the cytosolic NADH:NAD^+^ ratio is typically approximately 1:700; thus, changes in NAD^+^ levels due to shifts in this ratio are much smaller than changes in NADH levels. In addition to its effects on CtBP1, the increased NADH levels resulting from lactate oxidation may also activate NAD(P)H oxidase and contribute to ROS production.^45^ Further research is needed to ascertain whether the proinflammatory effect of lactate is dependent on this mechanism.

CtBP1, a transcriptional corepressor and metabolic sensor protein, interacts with transcription factors that contain a PXDLS‐like motif to suppress the transcription of target genes. Prior studies have shown that CtBP1 interacts with histone deacetylases, histone methyltransferases, Krueppel‐like factor 4, and p300 acetyltransferase.^20^, ^21^, ^46^ Our research broadens this understanding by revealing that CtBP1 also binds with FOXP1, which contains a potential PXDLS motif in its N‐terminal repression domain. FOXP1 transrepresses NLRP3 inflammasome components, and its targeted deletion in ECs intensifies atherosclerosis in *Apoe* ^−/−^ mice.^29^ We discovered that FOXP1 directly transrepresses *VCAM‐1* and *ICAM‐1*, thereby reducing endothelial inflammation. Moreover, our data on ECs treated with a synthetic peptide that inhibits CtBP1 oligomerization or ECs overexpressing an NADH‐insensitive CtBP1 demonstrated that only monomeric CtBP1 can bind with FOXP1 and enhance its ability to transrepress target genes. In contrast, when CtBP1 forms dimers in the presence of NADH, it is unable to bind with FOXP1, and its ability to enhance FOXP1's transrepressive effect on genes such as *VCAM‐1* and *ICAM‐1* is reduced. Our study revealed that the oligomerization status of CtBP1, which is affected by lactate, modulates FOXP1 activity in ECs. With endothelial‐specific KD of FOXP1, we found that lactate could accelerate atherogenesis through the regulation of FOXP1 activity, suggesting that the proinflammatory effects of lactate in vitro and the proatherogenic effects of lactate in vivo could be achieved, at least partially, through the same mechanisms. A previous study showed that lactate that accumulates in tumor tissues is taken up by ECs via MCT1 and induces tumor angiogenesis in a ROS‐ and NF‐κB/IL‐8‐dependent manner.^9^ Further research is needed to determine whether these events occur in the context of atherosclerosis and whether FOXP1 can synergize with NF‐κB to regulate endothelial inflammation.

Although previous studies have suggested that lactate may play a role in the development of atherosclerosis, direct evidence has been limited. Using endothelial‐targeting PEI nanoparticles containing si*MCT1* in vivo, our study revealed that the inhibition of endothelial MCT1 effectively mitigates atherosclerosis. Prior studies have indicated that suppression of lactate uptake in ECs by silencing *MCT1* ameliorates lactate‐promoted cardiac dysfunction after myocardial infarction.^10^ These findings underscore the potential of targeting endothelial MCT1 as a viable therapeutic strategy to combat atherosclerosis and prevent its consequent cardiovascular events. Furthermore, our findings indicate that LDH‐B‐mediated oxidation of lactate to pyruvate is crucial for lactate‐induced endothelial inflammation, suggesting that LDH‐B also represents a potential target for antiatherosclerosis therapy, meriting further investigation. Despite these strengths, our study has several limitations. First, although we employed si*MCT1*‐encapsulated endothelial‐targeting PEI nanoparticles to knock down endothelial MCT1, a genetic‐based approach involving endothelium‐specific gene knockout could further strengthen this study. Second, this research was conducted in male mice to minimize sex‐related variability because estrogen is known to have potential atheroprotective effects. Consequently, future experiments involving female mice are necessary. Finally, although PEI is well tolerated in mice, it is not approved by the Food and Drug Administration for human use. Future studies are needed to optimize the formulation of nanoparticles for clinical applications.

In summary, the present study reveals a new mechanism through which lactate is involved in vascular inflammation during atherosclerosis. Lactate modulates the transrepressive impact of FOXP1 on adhesion molecules through its effect on CtBP1 oligomerization, which is dependent on NADH. Lactate's harmful epigenetic signaling in vascular inflammation is facilitated by its influx through MCT1, suggesting the possibility of mitigating vascular inflammation by selectively targeting MCT1 activity in the context of atherosclerosis.

## 4 MATERIALS AND METHODS

### 4.1 Animal procedures

All procedures involving animals were given approval by the Institutional Animal Care and Use Committee at Guangzhou Medical University (approval number GY2022‐004) and were carried out in line with the recommendations specified in the Guide for the Care and Use of Laboratory Animals of the NIH guidelines. Information regarding the creation of atherosclerotic mice and their treatment can be located in *Materials and Methods* section of Supporting Information. For the purpose of anesthesia, mice were given intraperitoneal injections of ketamine (100 mg/kg body weight) and xylazine (10 mg/kg) (Phoenix Scientific, Inc., St. Joseph, MO, USA). The mice were euthanized using CO_2_ asphyxiation.

### 4.2 Human atherosclerosis

All procedures involving human participants were carried out in compliance with the principles set forth in the Declaration of Helsinki. Each participant provided informed consent. Specimens of the human carotid artery, encompassing both atherosclerotic and nonatherosclerotic segments, were gathered from carotid endarterectomy procedures. Additional details can be located in *Materials and Methods* section of Supporting Information.

### 4.3 Molecular methods and reagents

Detailed protocols for expression vectors, cell culture and transfection, isolation of mouse aortic ECs, immunoblotting, RT‐PCR, IP, luciferase reporter gene assay, glutaraldehyde cross‐linking, and the preparation and characterization of DSPE–PEG–PEI/Pep/siRNA nanoparticles, as well as immunostaining, are provided in Supporting Information. The CHIP assay, site‐directed mutagenesis, and quantification of NAD^+^, NADH, and L‐lactate concentrations were conducted using commercial kits, with specifics available in Supporting Information. Analysis of the scRNA‐seq dataset from atherosclerotic plaques was performed as outlined in Supporting Information.

### 4.4 Statistical analysis

Data are represented as the mean ± SEM. A two‐sided unpaired Student's *t*‐test was used for comparisons between two groups, while one‐way analysis of variance (ANOVA) with Tukey's *post hoc* test was employed for multiple comparisons. A *p*‐value less than 0.05 was deemed statistically significant. More details can be located in the Supplementary Material.

## AUTHOR CONTRIBUTIONS

Y. X., B. Y., and Z. L. designed the research. Z. L., S. G., K. C., Y. D., Y. Z., R. Y., Y. C., Z. L., S. H., M. C., and C. W. performed the research. Z. L., S. G., K. C., Y. D., Z. L., S. H., Y. Z., S. Y., Z. B., W. H., and J. H. analyzed the data. Y. X., B. Y., Z. L., and Y. D. wrote the manuscript. All authors have read and approved the final manuscript.

## CONFLICT OF INTEREST STATEMENT

The authors declare no conflicts of interest.

## ETHICS STATEMENT

All animal procedures were approved by the Institutional Animal Care and Use Committee at Guangzhou Medical University (approval number GY2022‐004). For procedures involving human subjects, written informed consent was obtained from all participants. Specimens of the human carotid artery obtained from carotid endarterectomy procedures were exempt from human subject review by the Institutional Review Board at Guangzhou Medical University, as they were classified as discarded human tissue.

## Supporting information



Supporting Information

## Data Availability

All data that support the findings in this study are available from the corresponding author upon reasonable request.
