# Supplementary material for: Endothelial monocarboxylate transporter 1 drives atherosclerosis via a lactate/NADH/CtBP‐mediated transrepression pathway
Source: MedComm (2020). 2025 Feb 13;6(2):e70089. doi: 10.1002/mco2.70089 (PMC11822463; doi:10.1002/mco2.70089)
Supplement: Supplementary file 1 — Supporting Information [file MCO2-6-e70089-s001.docx]

**Endothelial monocarboxylate transporter 1 drives atherosclerosis via a lactate/NADH/CtBP-mediated transrepression pathway**

Zou Li^1,2^^#^, Shuai Guo^2#^, Kaixiang Cao^2#^, Yuxi Duan^3^, Yuan Zhao^2^, Yuting Zhang^2^, Shihui Yu^2^, Zaixia Bai^2^, Runfa Yu^2^, Yixin Chen^2^, Ziling Li^2^, Shuqi Huang^2^, Mingchuan Song^2^, Cailing Wang^2^, Wenzhong Hou^4^, Jun He^5*^, Bin Yang^3*^, Yiming Xu^1,2*^

^1^Department of Emergency, The Second Affiliated Hospital, Guangzhou Medical University, Guangzhou, 510260, China

^2^School of Basic Medical Sciences, Guangzhou Medical University, Guangzhou, 511436, China;

^3^School of Biomedical Engineering, Guangzhou Medical University, Guangzhou, 511436, China.

^4^Department of Cerebrovascular Disease, The Affiliated Qingyuan Hospital (Qingyuan People’s Hospital), Guangzhou Medical University, Qingyuan, 511518, China.

^5^Department of Rehabilitation Center, The First Affiliated Hospital of Guangzhou University of Chinese Medicine, Guangzhou, 510080, China.

***Running title:*** Lactate drives endothelial inflammation

^#^Zou Li, Shuai Guo, and Kaixiang Cao contributed equally to this work.

***Correspondence:**

Yiming Xu, PhD

School of Basic Medical Sciences

Guangzhou Medical University

Phone: 0086-20-37105196 (Office)

Fax: 0086-20-37103099

Email: [xuyiming0807@gmail.com](mailto:xuyiming0807@gmail.com)

Bin Yang, PhD

School of Biomedical Engineering

Guangzhou Medical University

Email: [bin.yang@gzhmu.edu.cn](mailto:bin.yang@gzhmu.edu.cn)

Jun He, MD

The First Affiliated Hospital of Guangzhou University of Chinese Medicine

Phone: 0086-20-36591366 (Office)

Fax: 0086-20-36591595

Email: [hejunzj@gzucm.edu.cn](mailto:hejunzj@gzucm.edu.cn)

**Supplementary Materials and Methods**

**Mouse generation and breeding**

All animal studies were conducted with the approval of the Institutional Animal Care and Use Committee at Guangzhou Medical University (approval number GY2022-004) and in compliance with the Guide for the Care and Use of Laboratory Animals published by the US National Institutes of Health. Male C57BL/6 mice, aged eight weeks, were obtained from Beijing HFK Bioscience Co. LTD. For the atherosclerosis study, *Apoe*^-/-^ mice (Stock Number: 002052, The Jackson Laboratory) were fed a high-fat diet (HFD) consisting of 20% protein, 50% carbohydrate, 21% fat, and 0.21% cholesterol (Research Diets) for a period of 12 weeks. All mice were maintained on a 12:12-hour light-dark cycle with ad libitum access to water and standard pathogen-free chow.

**Human atherosclerosis**

All procedures involving human subjects adhered to the ethical standards set forth in the Helsinki Declaration. Informed consent was obtained from each participant prior to participation. Human carotid artery tissues, including both atherosclerotic and nonatherosclerotic segments, were procured from carotid endarterectomy procedures and subsequently stained for MCT1. These samples were exempt from human subject review by the Institutional Review Board at Guangzhou Medical University as they were classified as discarded human tissue. The expression of MCT1 in endothelium was assessed in both atherosclerotic and nonatherosclerotic arteries.

**Reagents**

AZD3965 (S7339, Selleck) was dissolved in DMSO and used at a final concentration of 500 nM for *in vitro* experiments. Lactate (L1750, Sigma) and MTOB (K6000, Sigma) were dispersed in PBS and used at final concentrations of 10 mM and 2.5 mM, respectively. Sodium pyruvate (ST1663, Beyotime Biotechnology) was obtained commercially and used at a final concentration of 10 mM. DSPE-PEG2000-MAL and DSPE-PEG2000-NHS were procured from Aladdin. Dimethylformamide (DMF) was obtained from J&K Scientific. Ethylene imine polymer (PEI, MW=10000) was acquired from Macklin. The CVHPKQHR peptide (Pep) was purchased from Dechi Biosciences Co., Ltd. GelRed was obtained from Vazyme Biotechnology Co., Ltd.

**Isolation of primary MAECs**

Primary mouse aortic endothelial cells (MAECs) were isolated from C57BL/6 and *Apoe*^-/-^ mice using a modified protocol previously described.^1^ Briefly, mice were euthanized via CO2 asphyxiation and the aorta was carefully dissected, with perivascular fat and adventitia removed. The isolated aorta was then washed three times with ice-cold Dulbecco’s Modified Eagle Medium (DMEM). A collagen gel was prepared by diluting type I collagen (BD Bioscience, Cat. No. 354236) with endothelial cell medium (ECM; Sciencell Research Laboratories, Cat. No. 1001) to a final concentration of 1.75 mg/ml. The diluted collagen gel was added to 24-well plates and incubated at 37°C for at least 30 minutes. The aortas were sectioned into small rings (~1 mm in length), opened, and placed onto the collagen gel with the endothelium facing the gel. After 3-5 days, when cellular outgrowth from the aortic segments was observed, the aortic rings were removed from the collagen. The matrix was digested with 1 mg/ml collagenase D (Sigma, Cat. No. 11088882001) for 10 minutes at 37°C. Following centrifugation, cells were reseeded in T25 tissue culture flasks and cultured in ECM at 37°C with 5% CO2.

**Cell culture and treatments**

Primary human umbilical vein endothelial cells (HUVECs) were isolated from umbilical cords using standard procedures in accordance with established guidelines. HUVECs at passages 3-8 and MAECs at passages 2-4 were cultured in endothelial cell medium (ECM; Sciencell Research Laboratories, Cat. No. 1001). In some experiments, the culture medium was supplemented with 10 mM lactate (adjust to pH 6.8; Sigma, Cat. No. L1750), 500 nM AZD3965 (Selleck, Cat. No. S7339), 10 mM pyruvate (Beyotime Biotechnology, Cat. No. ST1663), 50 nM AXKO-0046 dihydrochloride (GLPBIO, Cat. No. GC68710), or 2.5 mM MTOB (Sigma, Cat. No. K6000). The HEK293T cell line was cultured in DMEM supplemented with 10% fetal bovine serum at 37°C with 5% CO2.

**Protein extraction and western blotting**

HUVECs were lysed using RIPA buffer (Beyotime, Cat. No. P0013C) supplemented with 1% protease inhibitor cocktail (Beyotime, Cat. No. P1005) and 1% PMSF at 4°C for 30 minutes. Following centrifugation, protein concentrations in the cell lysates were determined using the BCA Protein Assay Kit (Thermo Fisher Scientific, Cat. No. 23235). A total of 20 μg of protein was separated by SDS-PAGE and transferred to PVDF membranes. The membranes were blocked and then incubated overnight at 4°C with primary antibodies against VCAM-1 (Cell Signaling Technology, Cat. No. 13662S; 1:2000), ICAM-1 (Santa Cruz Biotechnology, Cat. No. sc-8439; 1:1000), FOXP1 (Cell Signaling Technology, Cat. No. 4402S; 1:1000), MCT1 (Abcam, Cat. No. ab85021; 1:2000), HIS (Proteintech, Cat. No. 66005-1-IG; 1:2000), HA (Abcam, Cat. No. ab9110; 1:1000), FLAG (Sigma, Cat. No. F1804; 1:2000), and β-actin (Santa Cruz Biotechnology, Cat. No. sc-81178; 1:5000). Immunoreactive bands were visualized using the ChemiDoc MP system (Bio-Rad Laboratories) and band densities were quantified using Image Lab software (Bio-Rad Laboratories).

**Co-immunoprecipitation**

Following treatment, cells were washed twice with ice-cold PBS and lysed using RIPA buffer (Beyotime, Cat. No. P0013C) supplemented with 1% protease inhibitor cocktail (Beyotime, Cat. No. P1005) and 1% PMSF on ice for 30 minutes. After centrifugation, the supernatant was quantified and preincubated overnight at 4°C with target antibodies (anti-FLAG, anti-CtBP1, or anti-FOXP1). The mixture was then incubated with protein A/G Magbeads (Thermo Fisher Scientific, Cat. No. 88803) at 4°C for 3-4 hours. The antibody-conjugated protein G-Sepharose beads and target supernatant were incubated overnight. The protein-bead complex was washed three times with 0.25% Triton X-100/PBS. Bound proteins were eluted using 1×sample loading buffer and heated to 100°C for 5 minutes. Proteins were then analyzed by immunoblotting.

**Glutaraldehyde Cross-linking**

FLAG-tagged CtBP1-overexpressed HUVECs were subjected to either a vehicle or a 10 mM lactate solution for a duration of 3 h. Subsequently, the whole cell lysates, prepared using a 0.5% NP-40 buffer, were treated with freshly prepared glutaraldehyde at a working concentration of 0.25‰, maintained at a temperature of 37℃ for 5 min. The reaction was then halted by the addition of Tris-HCl (pH 8.0) at a working concentration of 50 mM. Finally, the modified samples were analyzed using Western blotting.

**Real-time PCR**

Total RNA was extracted from cultured cells or aortic endothelium using Trizol reagent (Invitrogen, Cat. No. 1596026). A total of 500 ng of RNA was used as a template for reverse transcription reactions with random hexamer primers using the iScript cDNA synthesis kit (Bio-Rad, Hercules, CA, USA). Quantitative PCR (qPCR) was performed on an ABI 7500 Real-Time PCR system (Applied Biosystems) using gene-specific primers listed in **Table S1**. Relative gene expression was calculated using the 2^−ΔΔct^ method with β-actin as an internal control. All experiments were independently repeated at least three times. Primers were synthesized and purified by Ige Biotechnology.

**RNA extraction from aortic endothelium**

As previously described,^2^ anesthetized mice were perfused with ice-cold PBS via the left ventricle and the thoracic aorta was carefully excised. Periadventitial tissues were removed and the aortas were transferred to a clean microfuge tube. The thoracic aorta was quickly flushed with 200 μL of TRIzol reagent (Thermo Fisher Scientific, Cat. No. 15596026) using a 29-gauge syringe. The TRIzol elution was used for RNA extraction to determine gene expression in mouse aortic endothelial cells.

**RNA interference**

HUVECs grown to 60-70% confluence in six-well plates were transfected with 25 nM siRNA using Lipofectamine RNAiMAX (Thermo Fisher Scientific) and OPTI-MEM (Thermo Fisher Scientific, Cat. No. 31985070) according to the manufacturer’s instructions. Six hours post-transfection, the medium was replaced with complete growth medium for continued culture. Cells were subjected to various treatments within 48 hours after siRNA transduction and then collected for downstream assays. The siRNA sequences targeting human *MCT1* (5′-AAGAGG CUGACUUUUCCAAAU-3′), mouse *Mct1* (5′- CCAAATCCATCACTGTCTT-3′), and scrambled siRNAs (5′-TTCTCCGAACGTGTCACGT-3′) were chemically synthesized by RiboBio.

**L-Lactate measurement**

The quantification of lactate levels in serum and aorta tissue samples was performed using the Lactic Acid (LA) Content Assay Kit (AKAC001M, Boxbio according to the manufacturer’s protocol. For serum samples, a 100μl aliquot was diluted in 1mL of lactate extract buffer A. For aorta tissue samples, the samples were processed according to their mass in grams, with a ratio of 1: (5-10) for lactate extract buffer A (mL). It is recommended to weigh 0.1 g of tissue and add 1 mL of lactate extract buffer A. The mixture was then subjected to centrifugation at 4℃ at a speed of 12000 g for 10 min. Subsequently, 800μL of the supernatant was collected into a new centrifuge tube, to which 150μL of extract liquid B was added and thoroughly mixed. This mixture was again centrifuged at 4℃ for 12000 g for 10 min. The supernatant was then collected and the corresponding buffer reaction was added as per the instructions provided with the kit. The absorbance of the resulting solution was measured using a microplate reader at 570 nm. The lactate content was then calculated based on the instructions provided with the kit.

**Quantification of NADH content and NADH/NAD^+^ ratio**

Intracellular NADH and NAD^+^ concentrations, as well as the NADH/NAD^+^ ratio, were determined using the NAD/NADH Assay Kit (Colorimetric; ab65348, Abcam; Cambridge, UK) according to the manufacturer’s protocol.

**Subclone and plasmid transfection**

CtBP1-FLAG/HA, containing the complete human CtBP1 coding sequence (NCBI Reference Sequence NM_001328.3) and an N-terminal FLAG/HA epitope, and FOXP1-HA, comprising the full-length human FOXP1 coding sequence (NCBI Reference Sequence NM_032682.6) and an N-terminal HA epitope, were generated by gene synthesis (IGE Biotechnology) and cloned in-frame into the pCDNA3.1-MCS-EFla-copGFP vector (Invitrogen). Human embryonic kidney (HEK) 293T cells (ATCC, Cat. No. CRL-1573) were transfected with vector plasmids using Lipofectamine 3000 (Thermo Fisher Scientific) at 60-70% confluence. Forty-eight hours post-transfection, cells were subjected to various treatments before being harvested for downstream assays.

**Site-directed mutagenesis**

Site-directed mutagenesis was performed using the QuickMutation™ Site-Directed Mutagenesis Kit (Beyotime, Cat. No. D0206S) to generate NADH-insensitive FLAG- or HA-tagged CtBP1 (G183A). The following primer pair was used to introduce the mutation: forward, 5’-GGCATCATCGGACTTGCTCGCGTGGGGCAGGCA-3’; reverse, 5’-CCGTAGTAGCCTGAACGAGCGCACCCCGTCCGT-3’. The mutation was confirmed by DNA sequencing.

**Adenoviral transduction**

HUVECs grown to 50% confluence were infected with recombinant adenovirus vectors (10 pfu/cell) harboring FLAG-tagged human CtBP1, His-tagged human CtBP1, HA-tagged human FOXP1, or a negative control adenovirus (Ad-Ctrl) for 6 hours. The medium was then replaced with fresh complete growth medium for continued culture. Thirty-six hours post-transduction, cells were used for downstream experiments.

**AAV-mediated EC-specific *Foxp1* silencing**

Recombinant adeno-associated virus serotype 9 (AAV9) vectors with a Cdh5 promoter carrying control shRNA (AAV-*Cdh5*-sh*Ctrl*) or shRNA against mouse *Foxp1* (AAV-*Cdh5*-sh*Foxp1*) were produced by Cyagen Biosciences Inc. *Apoe*^-/-^ mice were transduced with AAV-*Cdh5*-sh*Ctrl* or AAV-*Cdh5*-sh*Foxp1* (1×10^12^ genome-containing particles/mouse) via tail vein injection. Two weeks post-injection, high-fat Western diet-induced atherosclerosis was induced in *Apoe*^-/-^ mice as previously described.

**Chromatin immunoprecipitation (ChIP) assay**

ChIP assays were performed using the EZ-Magna ChIP^TM^ A/G kit (#17-10086; Millipore Sigma, Burlington, MA, USA). Briefly, HUVECs grown in 10 cm plates were rinsed three times with ice-cold PBS and fixed for 10 minutes with 1% formaldehyde. Fixation was quenched with glycine for 5 minutes at room temperature. Fixed HUVECs were washed three times with ice-cold PBS and lysed in lysis buffer containing protease inhibitor cocktail. DNA was sonicated to an average fragment size of 200-1000 base pairs. Following centrifugation at 15,000×g at 4°C for 15 minutes, the supernatant was incubated overnight at 4°C with protein A/G magnetic beads and anti-FOXP1 or pre-immune IgG. Unbound DNA fragments were removed from the immune complex-bound beads using low salt wash buffer, high salt wash buffer, LiCl wash buffer, and TE buffer in sequence. The DNA/protein complex was eluted using elution buffer. Crosslinks were reversed and chromatin was treated with proteinase K for 2 hours at 62°C with shaking. Immunoprecipitated DNA was purified using spin columns and subjected to PCR using primers listed in **Table S2**.

**Preparation of mouse aortas and quantification of atherosclerosis**

High-fat Western diet-induced atherosclerosis was induced in *Apoe*^-/-^ mice as previously described^3^. Aortas from atherosclerotic mice were collected and processed for Oil Red O staining of both *en face* preparations of whole aortas and cross-sections of aortic sinuses. Each section of the aortic sinus was 5 µm thick and sections were taken 48 µm apart, resulting in the analysis of six slides over a length of 288 µm of the aortic sinus. Six mice were included in each group. Images were scanned into a Macintosh computer and analyzed using ImageJ software (NIH). Analysis of en face Oil Red O staining was performed in a blinded manner.

**Immunofluorescence (IF) staining**

For *en face* staining, thoracic aortas were excised and adventitia was removed. Aortas were longitudinally sliced, fixed in 4% paraformaldehyde for 20 minutes at room temperature, and permeabilized with 0.5% Triton X-100 in PBS for 30 minutes. After blocking with 10% normal serum of the same species for 1 hour at room temperature, aortas were incubated overnight at 4°C with primary antibodies against CD31, ICAM-1, or MCT1. Tissues were then washed three times with PBS and incubated for 1 hour at room temperature with the appropriate Alexa Fluor 488 or Alexa Fluor 555-conjugated secondary antibodies diluted 1:500 in blocking solution. After washing three times with PBS, tissues were stained with DAPI diluted 1:10000 for 5 minutes.

For IF staining in mouse aortas and human carotid artery, 5 μm frozen or paraffin sections were cut and heated in TE buffer (pH 9.0) for 10 minutes for antigen retrieval. After permeabilization and blocking, sections were incubated overnight at 4°C with primary antibodies against VCAM-1, ICAM-1, or MCT1, followed by incubation with Alexa Fluor 594- and/or Alexa Fluor 488-labeled secondary antibodies. Slides were mounted using anti-fade mounting medium with DAPI (Beyotime) to visualize nuclei. Images were acquired using a Leica SP8 DMRB fluorescence microscope (Leica Microsystems). Antibody specificity and genuine target staining were validated by setting negative controls, which were incubated overnight with corresponding IgG and then stained with secondary antibodies.

**Luciferase reporter gene assay**

The promoter sequence of the *ICAM-1* gene, spanning from -1.6 to +0.1k and encompassing the potential wild-type or mutant FOXP1 binding sites, was individually subcloned into the pGL3 promoter luciferase vector. Similarly, the promoter sequence of the *VCAM-1* gene, ranging from -0.7 to +0.1k and containing the putative wild-type or mutant FOXP1 binding sites, was also independently subcloned into the pGL3 promoter luciferase vector. The final sequence of the plasmids was validated by DNA sequencing. Plasmids were transfected into HEK293T cells using Lipofectamine 3000 (L3000001, Life Technologies) according to the manufacturer’s instructions. Promoter luciferase activity was measured 36 hours post-transfection using the Dual-Luciferase assay kit (E1910; Promega, Madison, WI, USA). Individual luciferase activity was normalized to the corresponding renilla-luciferase activity.

**Single-Cell Gene Expression Analysis**

Single-cell RNA-sequencing (scRNA-seq) datasets from atherosclerotic plaques were obtained from the Gene Expression Omnibus database. These datasets were pooled from 11 *Ldlr*^-/-^ *LysMCre*^+/-^ mice (GSE150089). We employed the Seurat R package (v3.0.1) in R (v3.6.1) for our analysis. The single-cell gene expression was normalized by library size, scaled up by a factor of 10,000, and subsequently log-transformed. Our analysis was based on the original cell cluster annotations. The violin plots we created illustrate the normalized gene expression level for each cell cluster. Each data point corresponds to an individual observation (each cell), with the median of the distribution represented by a horizontal line. The number of cells, which constitutes the sample sizes, are indicated at the base of the plots.

**Synthesis of DSPE-PEG-Pep**

A solution of 20 mg DSPE-PEG2000-MAL was prepared by dissolving it in 5 mL of N,N-dimethylformamide (DMF). Subsequently, 5 mL of a DMF solution containing 20 mg of Pep was added to the mixture, which was stirred and allowed to react for 24 hours at room temperature. The resulting reaction solution was placed in a dialysis bag (MWCO = 3500 Da) and dialyzed against pure water for 2 days. The dialysate was collected and lyophilized to obtain the final product, DSPE-PEG-Pep.

**Synthesis of DSPE-PEG-PEI**

DSPE-PEG2000-NHS and PEI10000 were reacted in a 1:1 molar ratio. Specifically, DSPE-PEG2000-NHS and PEI10000 were separately dispersed in anhydrous DMF using ultrasonication, and the resulting solutions were stirred for 24 h under an argon atmosphere. The reaction mixture was then dialyzed for 2 days using a dialysis membrane with a molecular weight cut-off of 3500 Da. After lyophilization of the dialysate, the solid product was collected.

**Preparation of DSPE-PEG-PEI/Pep complexes**

DSPE-PEG-PEI and DSPE-PEG-Pep were combined in a 2:1 molar ratio and dispersed in 20 mL of deionized water using ultrasonication. The resulting mixture was stirred at room temperature for 24 h, and the complex solution was stored at 4°C until further use.

**Preparation of** **DSPE-PEG-PEI/Pep/siRNA** **nanoparticles**

A solution of siRNA was prepared at a concentration of 0.02 nmol/μL and incubated with DSPE-PEG-PEI/Pep at 37°C for 30 minutes at a specific N/P ratio. The mixture was then diluted with ultra-pure water to the desired volume for use.

**Characterization of** **DSPE-PEG-PEI/Pep/siRNA nanoparticles**

The structures of DSPE-PEG-Pep, DSPE-PEG-PEI, and the DSPE-PEG-PEI/Pep complex were characterized using proton nuclear magnetic resonance (1H NMR, JNM-EC Z400S/L1) and Fourier transform infrared spectroscopy (FTIR, TENSOR 27). The morphology of the DSPE-PEG-PEI/Pep/siRNA nanoparticles was examined using transmission electron microscopy (TEM, JEM-1400PLUS). The hydrodynamic size distribution and zeta potential of the DSPE-PEG-PEI/Pep/siRNA and the DSPE-PEG-PEI/siRNA nanoparticles were determined using dynamic light scattering (DLS, ZETASIZER NANO ZS) at 25°C.

**Agarose gel electrophoresis assay**

The siRNA binding affinity of DSPE-PEG-PEI and DSPE-PEG-PEI/Pep was evaluated using agarose gel electrophoresis. Samples were prepared by mixing the compounds with siRNA at a specified N/P ratio and incubating the mixture at 37°C for 30 minutes. The samples were then loaded into individual wells of an agarose gel stained with GelRed and electrophoresed at 100V for 20 minutes.

**Evaluation of siRNA encapsulation efficiency (EE)**

The amount of siRNA was quantified using a calibration curve constructed from solutions of known siRNA concentrations. The encapsulation efficiency of siRNA at different N/P ratios was calculated using the following formula: EE (%) = Nencapsulated siRNA/Ntotal siRNA × 100%, where Nencapsulated siRNA and Ntotal siRNA represent the amount of encapsulated siRNA and the initial amount of added siRNA, respectively. The encapsulation efficiency of siRNA is listed in **Table S3.**

**Serum stability**

The serum stability of siRNA and DSPE-PEG-PEI/Pep/siRNA nanoparticles in aqueous solution was analyzed using agarose gel electrophoresis. Samples were prepared by mixing the compounds with fetal bovine serum (FBS) at a 1:1 volume ratio to achieve a final serum concentration of 50% and incubating the mixture at 37°C. At various time points, samples containing siRNA were loaded onto agarose gels and subjected to electrophoresis.

***In vitro* cytotoxicity**

HUVEC cells were seeded in 96-well plates at a density of 5000 cells per well. After 24 h of incubation, the cells were treated with DSPE-PEG-PEI/siRNA and DSPE-PEG-PEI/Pep/siRNA at various N/P ratios. After an additional 24 h, 10 μL of MTT solution (5 mg/mL) was added to each well and incubated for 4 h. The medium was then replaced with 150 μL of DMSO, and the optical density (OD) was measured at a wavelength of 570 nm using a microplate reader.

**Statistical analysis**

Data were analyzed using GraphPad Prism 8 software (GraphPad Software). Differences between two groups were assessed using an unpaired Student’s *t*-test. For multiple comparisons, one-way analysis of variance (ANOVA) followed by Tukey’s *post hoc* test was used. Results are presented as mean ± standard error of the mean (SEM). A *p*-value < 0.05 was considered statistically significant (**p* < 0.05, ***p* < 0.01, ****p* < 0.001, *****p* < 0.0001).

1. Wang JM, Chen AF, Zhang K. Isolation and Primary Culture of Mouse Aortic Endothelial Cells. *Journal of visualized experiments : JoVE*. 12 19 2016;(118)

2. Yang Q, Xu J, Ma Q, et al. PRKAA1/AMPKα1-driven glycolysis in endothelial cells exposed to disturbed flow protects against atherosclerosis. *Nature communications*. 11 7 2018;9(1):4667.

3. Xu Y, Wang Y, Yan S, et al. Regulation of endothelial intracellular adenosine via adenosine kinase epigenetically modulates vascular inflammation. *Nature communications*. Oct 16 2017;8(1):943.

**Supplementary Figures
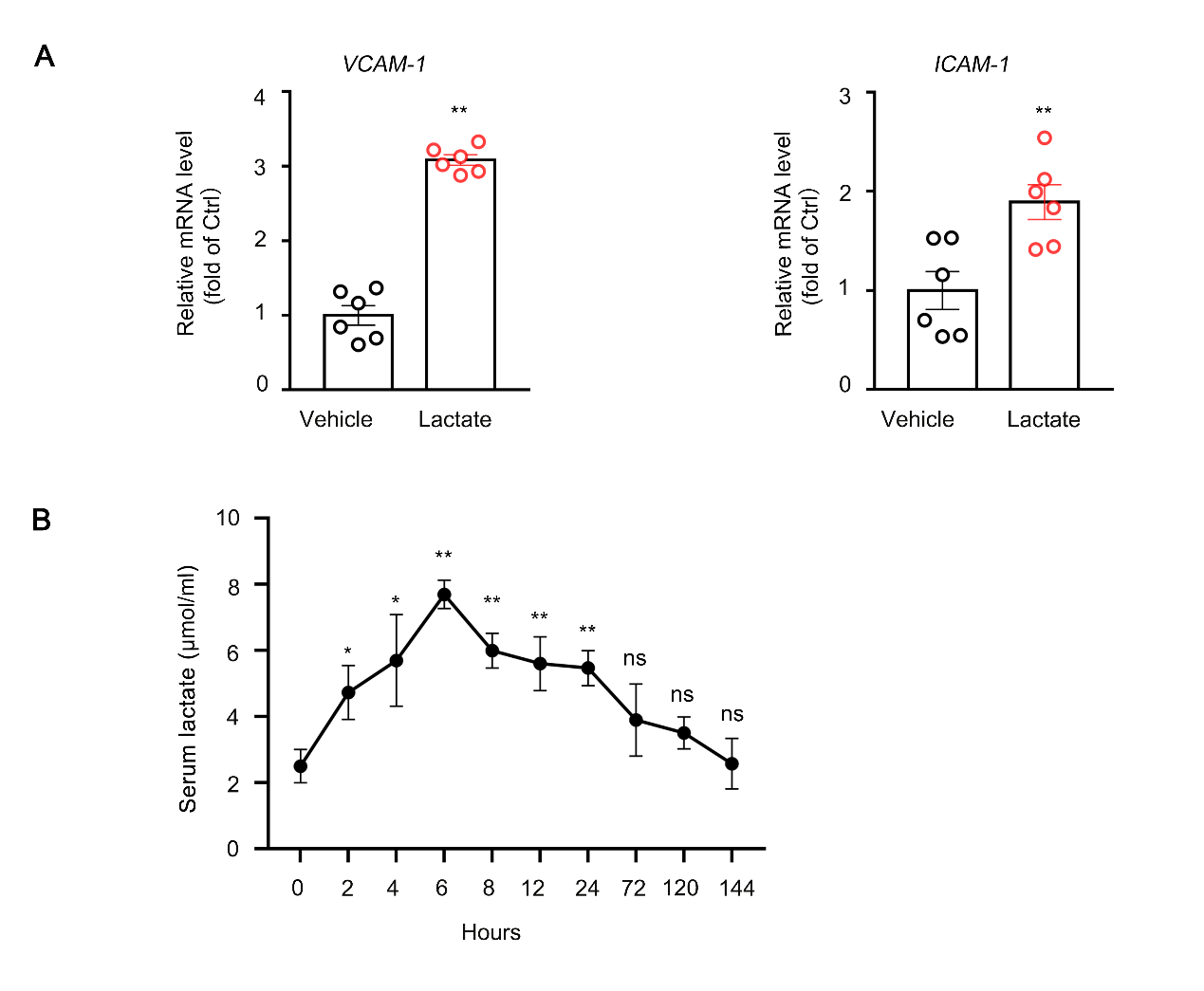
Figure S1. Lactate upregulated the mRNA levels of *VCAM-1* and *ICAM-1*.**

**(A)** Real-time PCR analysis of the mRNA levels of *VCAM-1* and *ICAM-1* genes in HUVECs treated with 10 mM lactate or vehicle for 3 h (n = 6). **(B)** The serum lactate levels in one-month WD-fed *Apoe*^-/-^ mice treated with lactate (0.5 g/kg body weight) via *i.p.* injection for 2, 4, 6, 8, 12, 24, 72, 120, and 144 h (n=3 mice per group). For all bar graphs, data are the mean ± SEM. Statistical significance was assessed using an unpaired, two-tailed Student’s *t*-test (A) or one-way ANOVA with Tukey’s *post hoc* test (B). Compared with the vehicle group, ***P* < 0.01 (A). Compared with the 0-hour group, **P* < 0.05 and ***P* < 0.01 (B).

**
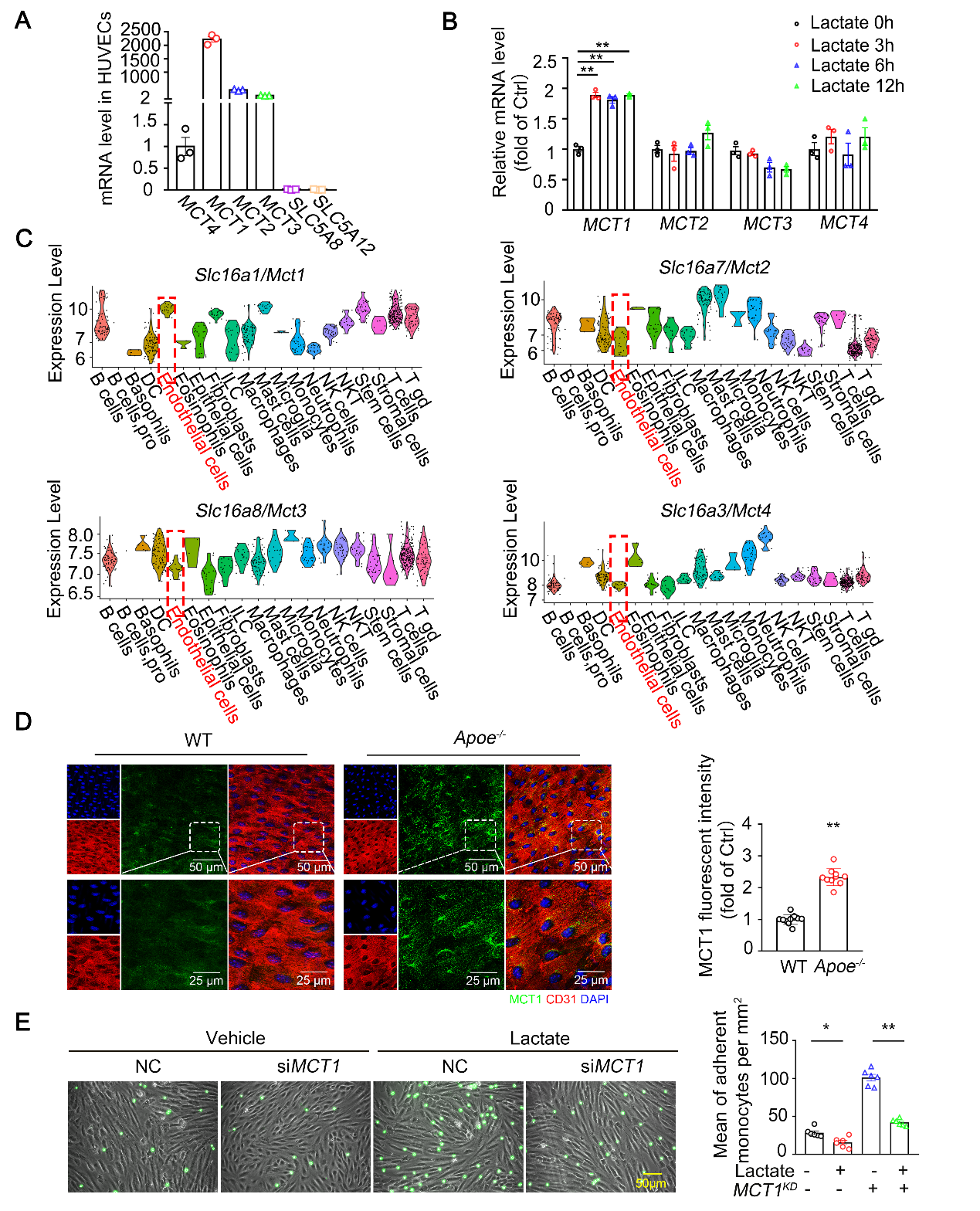
 Figure S2. Knockdown of MCT1 significantly decreased the expression of *VCAM-1* and *ICAM-1* in** **ECs.**

**(A)** Real-time PCR analysis of the mRNA levels of *MCT1*, *MCT2*, *MCT3*, *MCT4*, *SLC5A8*, and *SLC5A12* in HUVECs (n=3). **(B)** Real-time PCR analysis of the mRNA levels of *MCT1*, *MCT2*, *MCT3*, and *MCT4* in HUVECs treated with 10 mM Lactate or vehicle for 0-12 h (n=3). **(C)** Violin plot of *Mct1*, *Mct2*, *Mct3*, and *Mct4* expression in single-cell populations of murine *Ldlr*^-/-^ aortic arch lesions. **(D)** *En face* IF staining and quantification of Mct1 (green) in arterial endothelium of WT and *Apoe^-/-^* mice. The endothelium was visualized by CD31 staining (red). Nuclei were counterstained with DAPI (blue) (n=10 mice per group). **(E)** Representative images and quantification of monocyte adhesion on *MCT1* KD or control HUVECs treated with lactate at 10 mM for 6 h (n=6). For all bar graphs, data are the mean ± SEM. Statistical significance was assessed using one-way ANOVA with Tukey’s *post hoc* test (B, E) or an unpaired, two-tailed Student’s *t*-test (D). Compared with the lactate 0 h group, ***P* < 0.01 (B). Compared with the WT group, ***P* < 0.01 (D). The composition of the two groups delineated by horizontal lines, **P* < 0.05 and ***P* < 0.01 (E).

**
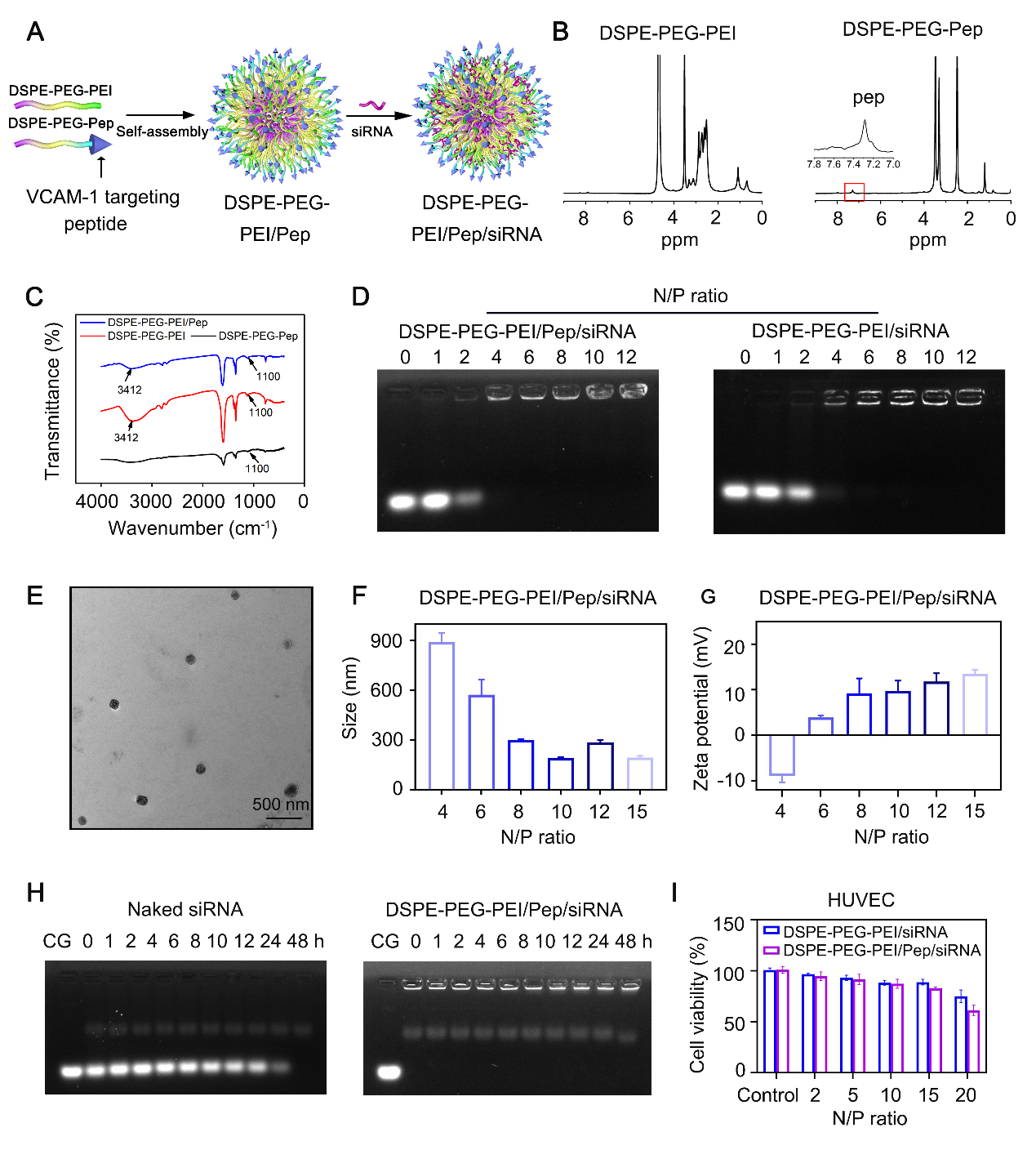
 Figure S3.** **Synthesis of DSPE-PEG-PEI, DSPE-PEG-Pep, and DSPE-PEG-PEI/Pep complexes and physicochemical characterization of DSPE-PEG-PEI/Pep/siRNA** **nanoparticles.**

**(A)** Schematic diagram of construction of DSPE-PEG-PEI/Pep/siRNA nanoparticles. **(B)** ^1^H NMR spectrum of DSPE-PEG-PEI and DSPE-PEG-Pep. **(C)** FTIR spectra of DSPE-PEG-PEI, DSPE-PEG-Pep, and the DSPE-PEG-PEI/Pep complexes. **(D)**  Agarose gel electrophoresis results for DSPE-PEG-PEI/siRNA and DSPE-PEG-PEI/Pep/siRNA at various N/P ratios. **(E)** TEM image of DSPE-PEG-PEI/Pep/siRNA nanoparticles at an N/P ratio of 15. **(F)** Particle size distribution of DSPE-PEG-PEI/Pep/siRNA nanoparticles at N/P ratios ranging from 4 to 15 (n=4). **(G)** Zeta potential of DSPE-PEG-PEI/Pep/siRNA nanoparticles at N/P ratios ranging from 4 to 15 (n=4). **(H)** The serum stability of siRNA and DSPE-PEG-PEI/Pep/siRNA nanoparticles in aqueous solution for 48 h. **(I)** *In vitro* cytotoxicity of DSPE-PEG-PEI/siRNA and DSPE-PEG-PEI/Pep/siRNA at different N/P ratios in HUVECs (n=4). For bar graphs, data are the mean ± SEM. Statistical significance was assessed using two-way ANOVA with Tukey’s *post hoc* test (I).

**
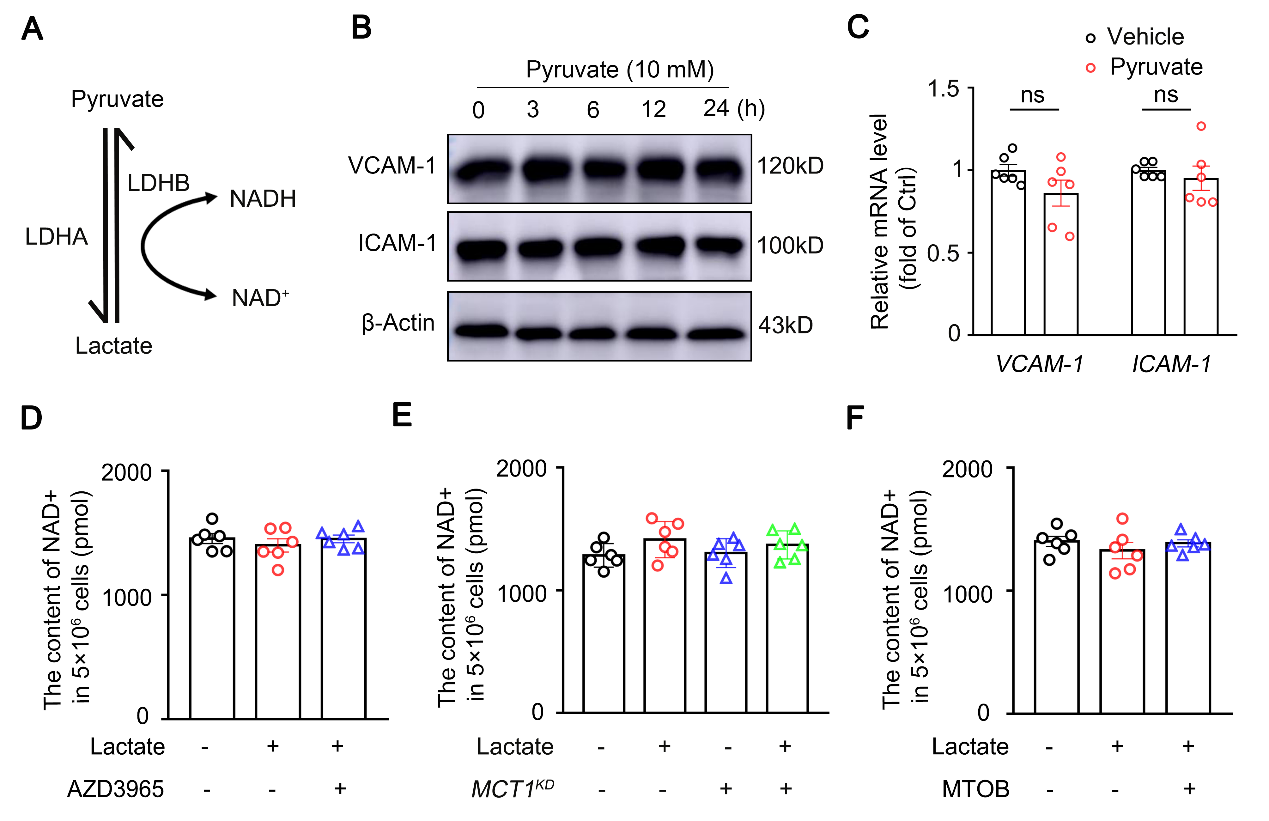
 Figure S4.** **Pyruvate did not induce endothelial inflammation.**

**(A)** Schematic diagram depicting the conversion of lactate to pyruvate, thereby regenerating NADH from NAD^+^. **(B)** Western blot detection of VCAM-1 and ICAM-1 in HUVECs treated with 10 mM pyruvate for 0-24 h. **(C)** Real-time PCR analysis of the mRNA levels of *VCAM-1* and *ICAM-1* in HUVECs treated with 10 mM pyruvate or vehicle for 3 h (n=6). **(D)** The content of NAD^+^ in lactate-treated HUVECs pretreated with the MCT1 inhibitor AZD3965 (500 nM for 24h) (n=6). **(E)** The content of NAD^+^ in lactate-treated *MCT1* KD or control HUVECs (n=6). **(F)** The content of NAD^+^ in lactate-treated ECs pretreated with the NADH depletor MTOB (2.5 mM for 24h) (n=6). For all bar graphs, data are the mean ± SEM. Statistical significance was assessed using an unpaired, two-tailed Student’s *t*-test (C) or one-way ANOVA with Tukey’s *post hoc* test (D, E, F). Compared with the vehicle group, ns indicates no significance (C).

**
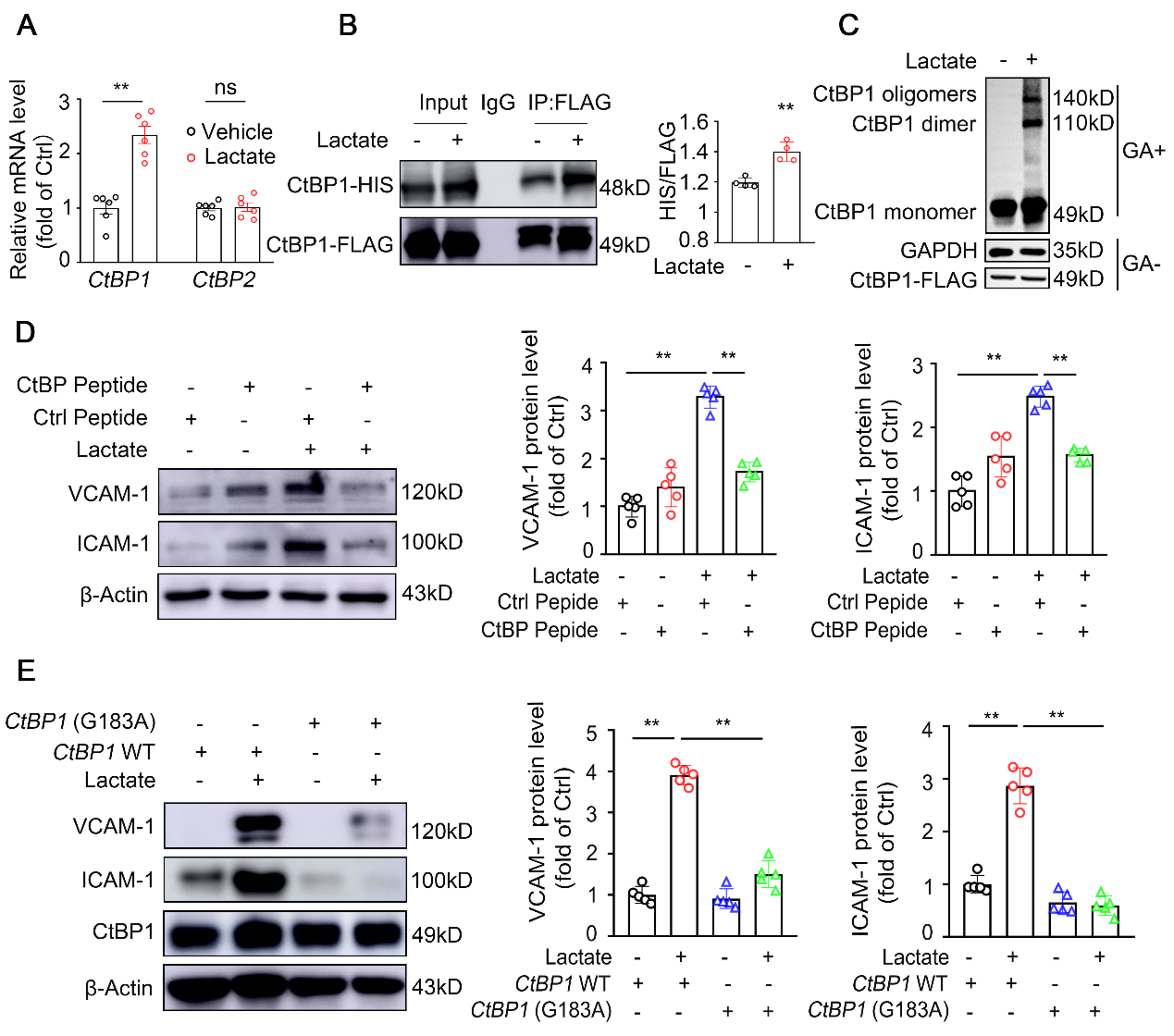
Figure S5.** **NADH-sensitive CtBP1 oligomerization is critical in endothelial activation.**

**(A)** Real-time PCR analysis of the mRNA levels of *CtBP1* and *CtBP2* genes in HUVECs treated with 10 mM lactate or vehicle for 3 h (n=6). **(B)** Co-IP of CtBP1-FLAG and CtBP1-HIS in FLAG/HIS-tagged CtBP1-overexpressed HUVECs treated with 10 mM lactate (n=4). **(C)** Change of CtBP1 oligomer states in FLAG-tagged CtBP1-overexpressed HUVECs treated with vehicle or 10 mM lactate for 3 h. The whole-cell lysates were treated with 0.25‰ glutaraldehyde (GA) for 5 min and then analysed by Western blot with the Flag antibody (n=3). **(D)** Western blot detection and quantification of the protein levels of VCAM-1 and ICAM-1 in HUVECs pretreated with Ctrl or CtBP peptide (20 µm) for 30 min and then stimulated with lactate (10 mM) or vehicle for 6 h (n=5). **(E)** Western blot detection and quantification of the protein levels of VCAM-1 and ICAM-1 in FLAG/HA-tagged WT CtBP1 or FLAG/HA-tagged G183A CtBP1-overexpressed HUVECs stimulated with 10 mM lactate or vehicle for 3 h (n=5). For all bar graphs, data are the mean ± SEM. Statistical significance was assessed using an unpaired, two-tailed Student’s *t*-test (A, B) or one-way ANOVA with Tukey’s *post hoc* test (D, E). Compared with the vehicle group, ***P* < 0.01 and ns indicates no significance (A, B). The composition of the two groups delineated by horizontal lines, ***P* < 0.01 (D, E).

**
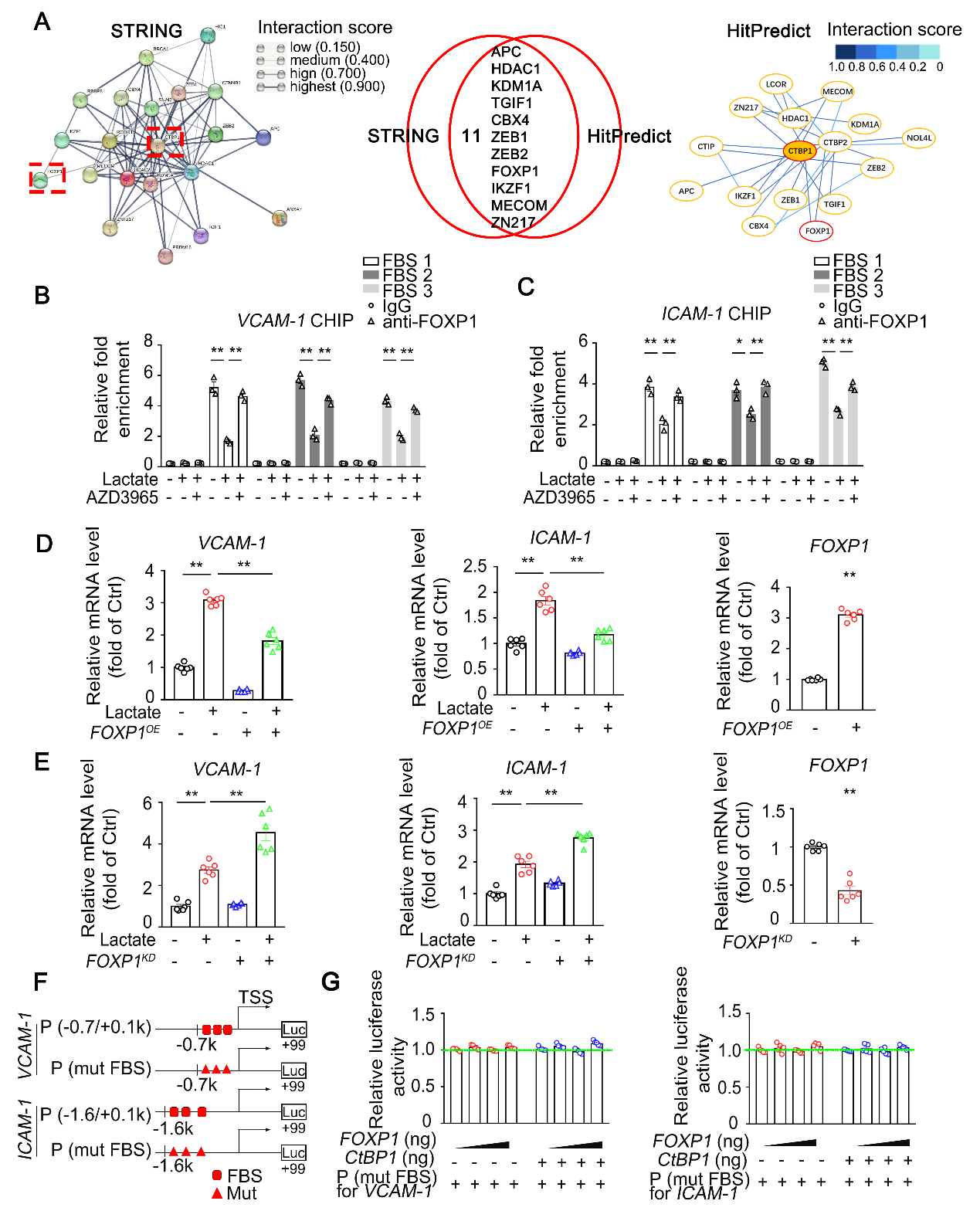
Figure S6.** **FOXP1 negatively regulates the transcription levels of VCAM-1 and ICAM-1.**

**(A)** The STRING and HitPredict databases showing the potential hits that might associate with human CtBP1. **(B and C)** ChIP analysis with antibodies against FOXP1 or IgG, soluble chromatin (≈500 bp in length) from HUVECs treated with vehicle or lactate (10 mM) after the MCT1 inhibitor AZD3965 (500 nM for 24 h) pretreatment, and primers targeting the region spanning all the consensus FOXP1 binding sites in the promoter region of *VCAM-1* and *ICAM-1* genes (n=3). **(D)** Real-time PCR analysis of the mRNA levels of *VCAM-1* and *ICAM-1* genes in FOXP1-overexpressed or control HUVECs stimulated with 10 mM lactate or vehicle for 3 h (n=6). **(E)** Real-time PCR analysis of the mRNA levels of *VCAM-1* and *ICAM-1* genes in *FOXP1* KD or control HUVECs stimulated with 10 mM lactate or vehicle for 3 h (n=6). **(F)** Schematic diagram of ﬁreﬂy luciferase reporters carrying the specified DNA fragments of the *VCAM-1* and *ICAM-1* promoters. Arrow indicates the transcription direction of *VCAM-1* and *ICAM-1*. Square (□): the putative FOXP1 binding sites (FBS). Triangle (Δ): the mutated FBS (mutFBS). TSS, transcriptional start site. Luc, luciferase gene. **(G)** Luciferase reporter assay showing the activity of *VCAM-1* and *ICAM-1* promoter regions containing the mutant putative FOXP1 binding sites (FBS) in FOXP1 and CtBP1-overexpressed HEK293T cells (n=5). For all bar graphs, data are the mean ± SEM. Statistical significance was assessed using one-way ANOVA (B, C, D, E) or two-way ANOVA (G) with Tukey’s *post hoc* test. The composition of the two groups delineated by horizontal lines, **P* < 0.05 and ***P* < 0.01 (B, C, D, E).

**
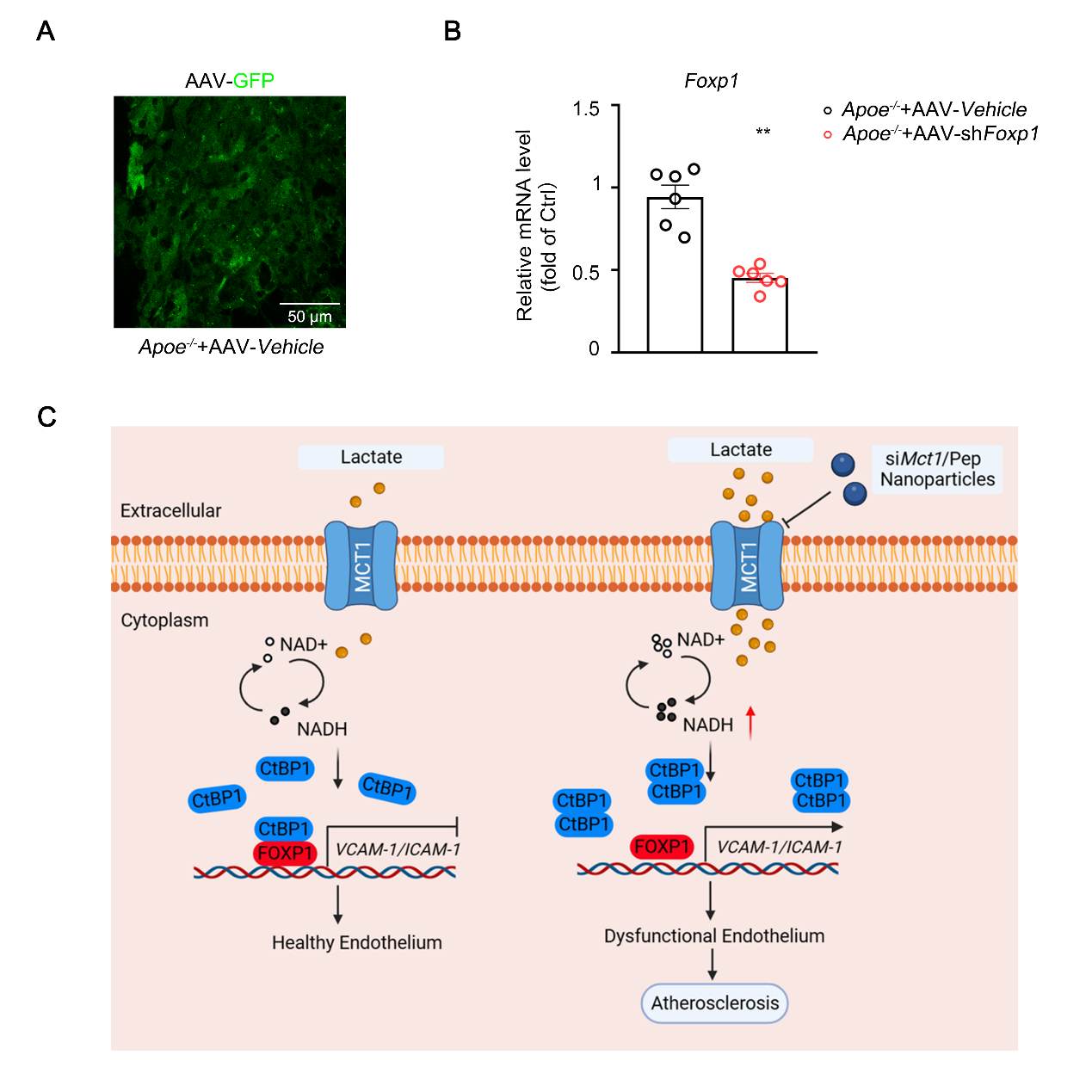
Figure S7. The efficiency of endothelial-specific *Foxp1* silencing.**

**(A)** Representative confocal image of intracellular GFP fluorescence in endothelium layer from *Apoe*^-/-^ mice treated with AAV-*Cdh5*-GFP. **(B)** Real-time PCR analysis of the mRNA level of *FOXP1* gene in endothelial RNA obtained from 4-week HFD-fed *Apoe*^-/-^ mice injected with AAV-*Cdh5*-CTRL and AAV-*Cdh5*-*shFXOP1*. Endothelial RNA was extracted from mouse aortas through flushing with TRIzol reagent (n=6 mice per group). **(C)** In quiescent ECs, CtBP1 binds to FOXP1, enhancing the transcriptional inhibition of VCAM-1 and ICAM-1 by FOXP1 and maintaining the quiescent state of the ECs. However, in the context of atherosclerosis, an excess of lactate in the tissue microenvironment can enter ECs via MCT1. During the conversion of lactate to pyruvate, a large amount of NADH is generated, promoting the oligomerization of NADH-sensitive CtBP1. The dimerized CtBP1 dissociates from FOXP1, partially relieving the transcriptional inhibition of VCAM-1 and ICAM-1 by FOXP1. This results in the upregulation of VCAM-1 and ICAM-1 expression, leading to endothelial inflammation and the development of atherosclerosis. For all bar graphs, data are the mean ± SEM. Compared with the *Apoe*^-/-^+AAV-vehicle group, ***P* < 0.01; unpaired, two-tailed Student’s *t*-test (B).

**Supplementary Tables**

**Table S1. Primer sequences for qPCR**

| Human Gene | Primer sequence (5’-3’) |
| --- | --- |
| *MCT1* | F: TGTTGACATGGTAGCCCGAC |
|  | R: CAACGGAAGCCGCAAAGAAA |
| *ICAM-1* | F: GGCCGGCCAGCTTATACAC |
|  | R: TAGACACTTGAGCTCGGGCA |
| *VCAM-1* | F: TCAGATTGGAGACTCAGTCATGT |
|  | R: ACTCCTCACCTTCCCGCTC |
| *CtBP1* | F: TCACAGGCCGGATCCCAGACAG |
|  | R: GGTACCTATAGGCAGCCCCATTGAGC |
| *FOXP1* | F: CGAATGTTTGCTTACTTCCGACGC |
|  | R: ACTTCATCCACTGTCCATACTGCC |
| *MCT2* | F: AGATGGAGGATGGGGTTGGA |
|  | R: TGCGTACATAACAGCCAGCA |
| *MCT3* | F: ACAGCCTGGATCTCCTCCAT |
|  | R: ATGATGCTCCGGCAAAAGGA |
| *MCT4* | F: GTGAATGTGTTTGTGATGGGGA |
|  | R: AAAGCAGAACCCAAACCTGC |
| *SLC5A8* | F: AACCAATCCCAGGTGCAGAG |
|  | R: GATTGCCCAGAGTCCCACAA |
| *SLC5A12* | F: GGGGGAAGGCAAATGAGCTT |
|  | R: AGATGACCGTGGCAGCATAG |
| *β-ACTIN* | F: TCACCCACACTGTGCCCATCTACGA |
|  | R: CAGCGGAACCGCTCATTGCCAATGG |
| Mouse Gene | Primer sequence (5’-3’) |
| *Mct1* | F: GCCGGAGTCTTTGGATTTGC |
|  | R: GGCAGCATTCCACAATGGTC |
| *Icam-1* | F: AAACCAGACCCTGGAACTGCAC |
|  | R: GCCTGGCATTTCAGAGTCTGCT |
| *Vcam-1* | F: GCTATGAGGATGGAAGACTCTGG |
|  | R: ACTTGTGCAGCCACCTGAGATC |
| *β-actin* | F: CTCTGGCTCCTAGCACCATGAAGA |
|  | R: GTAAAACGCAGCTCAGTAACAGTCCG |

**Table S2. Primer sequences for CHIP**

| Promoter | Primer sequence (5’-3’) |
| --- | --- |
| *VCAM-1* Primer1 | F: AGCAATTTCAGTTACAGTCC |
|  | R: AGGGACACCATAACTTCTTA |
| *VCAM-1* Primer2 | F: GAAGTTATGGTGTCCCTTTT |
|  | R: TACTCTGGTTTTTGAACTGG |
| *VCAM-1* Primer3 | F: GTCTCCATTTTTTCTCTCCCC |
|  | R: TTTCCTCATCTTCGACTCCA |
| *ICAM-1* Primer1 | F: TGTGACACCTCCCCTCAACT |
|  | R: CATAGGCTCACAACACCACA |
| *ICAM-1* Primer2 | F: ACAGAGCGAGAGCTTGTCTA |
|  | R: GTCCTGTTTGCATTTCACTG |
| *ICAM-1* Primer3 | F: ATGTGATTAGGACTGGGAGC |
|  | R: GTAGCATCAAAACACAAAGG |

**Table S3. Measurement of siRNA EE by Agarose gel Assay. Data were expressed as mean ± SD (n=3).**

| DSPE-PEG-PEI/siRNA | | DSPE-PEG-PEI/Pep/siRNA | |
| --- | --- | --- | --- |
| N/P | siRNA EE (%) | N/P | siRNA EE (%) |
| 0 | 0 | 0 | 0 |
| 1 | 10.15 ± 0.18 | 1 | 8.15 ± 1.07 |
| 2 | 33.71 ± 4.43 | 2 | 75.68 ± 5.94 |
| 4 | 85.59 ± 7.03 | 4 | 98.66 ± 0.41 |
| 6 | 97.96 ± 1.81 | 6 | 99.63 ± 0.64 |
| 8 | 98.60 ± 1.72 | 8 | 99.42 ± 0.44 |
| 10 | 99.45 ± 0.69 | 10 | 99.67 ± 0.08 |
| 12 | 99.69 ± 0.28 | 12 | 99.70 ± 0.46 |
